# Interpretable Differential Abundance Signature (iDAS)

**DOI:** 10.1002/smtd.202500572

**Published:** 2025-05-27

**Authors:** Lijia Yu, Yingxin Lin, Xiangnan Xu, Pengyi Yang, Jean Y. H. Yang

**Affiliations:** ^1^ School of Mathematics and Statistics The University of Sydney Camperdown NSW 2006 Australia; ^2^ Sydney Precision Data Science Centre University of Sydney Camperdown NSW 2006 Australia; ^3^ Charles Perkins Centre The University of Sydney Camperdown NSW 2006 Australia; ^4^ Laboratory of Data Discovery for Health Limited (D24H) Science Park Hong Kong SAR China; ^5^ Department of Biostatistics Yale University New Haven CT 208034 USA; ^6^ School of Business and Economics Humboldt‐Universität zu Berlin 10099 Berlin Germany; ^7^ Computational Systems Biology Unit Children's Medical Research Institute Faculty of Medicine and Health University of Sydney Westmead NSW 2145 Australia

**Keywords:** ANOVA, bioinformatics, differential abundance, differential expression, single cell

## Abstract

Single‐cell technologies have revolutionized the understanding of cellular dynamics by allowing researchers to investigate individual cell responses under various conditions, such as comparing diseased versus healthy states. Many differential abundance methods have been developed in this field, however, the understanding of the gene signatures obtained from those methods is often incomplete, requiring the integration of cell type information and other biological factors to yield interpretable and meaningful results. To better interpret the gene signatures generated in the differential abundance analysis, iDAS is developed to classify the gene signatures into multiple categories. When applied to melanoma single‐cell data with multiple cell states and treatment phenotypes, iDAS identified cell state‐ and treatment phenotype‐specific gene signatures, as well as interaction effect‐related gene signatures with meaningful biological interpretations. The iDAS model is further applied to a longitudinal study and spatially resolved omics data to demonstrate its versatility in different analytical contexts. These results demonstrate that the iDAS framework can effectively identify robust, cell‐state specific gene signatures and is versatile enough to accommodate various study designs, including multi‐factor longitudinal and spatially resolved data.

## Graphical abstract

iDAS is an ANOVA‐based framework that enhances the interpretability of differential abundance signatures in single‐cell and spatial omics data. It provides a coherent and interpretable framework for grouping gene‐level associations in complex experimental designs. Applications to melanoma immunotherapy datasets and spatial transcriptomics demonstrate iDAS's flexibility, robustness, and translational potential across diverse study designs.

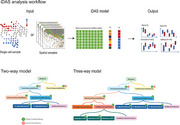

## 1 Introduction

The recent increase in multi‐sample and multi‐condition single‐cell studies offers significant potential to uncover cell types and molecular pathways crucial for the onset, progression, and treatment of diseases.^[^ ^1^, ^2^, ^3^ ^]^ These studies enable researchers to identify and analyze phenotype‐specific subpopulations and marker genes associated with various conditions. The standard approach to achieving this generally involves two steps. The first step consists in the identification of subgroups of cells that change in abundance in response to a given phenotype (e.g., Milo, DA‐seq). The second step consists in annotating these subpopulations by identifying markers that characterize these groups. Together, this enables scientists to reach certain conclusions regarding cell states and their corresponding markers that drive these changes.

Recently, a number of algorithms have been developed to analyze changes in cell populations in response to disease progression or experimental interventions. Notable methods include Milo,^[^ ^4^ ^]^ CNA,^[^ ^5^ ^]^ DA‐seq,^[^ ^6^ ^]^ MELD,^[^ ^7^ ^]^ and Scissor.^[^ ^8^ ^]^ Most of these methods begin by constructing a KNN graph and summarizing it into an abundance matrix. Statistical methods such as generalized linear models, logistic ridge regression, and kernel density estimation are then applied to link this matrix with sample‐level phenotype labels, ultimately identifying phenotype‐specific subpopulations. This is achieved by providing perturbation scores representing the significance of differential abundance (DA) tests or probabilities related to the phenotype. It is important to note that while many of these methods provide a “differential abundance score” associated with each cell, further analytics is often required to generate interpretable stories. Thus, a common next step involves selecting distinct signatures of these differentially abundant groups for more detailed analysis, typically using classic differential expression analysis methods. Here, the differential expression (DE) analysis can be approached from two levels. The first is the sample‐level view, where expression is aggregated to create “pseudobulk” samples. Following this, genes associated with sample‐level phenotype outcomes are identified using methods originally designed for bulk RNA samples, such as edgeR,^[^ ^9^ ^]^ DEseq2^[^ ^10^ ^]^ or Limma‐voom.^[^ ^11^, ^12^ ^]^ The second is the single cell level view, where differential expression (DE) analysis is done without creating “pseudobulk,” and cells are modeled individually using generalized mixed effects models, such as MAST^[^ ^13^ ^]^ or glmmTMB.^[^ ^14^ ^]^ Together, these approaches help understand the gene features most associated with or affected by pathological or experimental conditions, providing valuable insights into the underlying biological processes.

Although these workflows do not usually require cell type annotation in the initial differential abundant groups identification, the practical reality is that subsequent analytics often become complex as it requires results to align or account for cell types or other related factors for meaningful interpretation. For instance, many DA analysis methods are clustering‐free, allowing them to detect DE genes between differential abundance regions, which may include multiple cell types or experimental conditions, thus complicating the interpretation of these DE genes. To make these genes more interpretable, it is critical to jointly examine the effects of cell types and other potentially related factors.

To this end, we developed iDAS, a computational framework to identify signatures generated from differential abundance algorithms, while considering the effects of cell types and other potentially relevant factors. This is an “ANOVA”‐based model, where we identify the gene signatures in the subpopulation groups identified by the DA test. This can be thought of as a phenotype guided feature selection strategy where a model was used to assess whether gene signatures exhibit significant responses to multiple factors of interest. For example, iDAS aims to separate biomarkers or biosignatures that are common to all individuals or only specific to a given cell type. We believe that this analytical approach will improve the interpretability and clarity of data analysis by better classifying the signal from the data, thereby leading to a better understanding of the relation between gene signature and different factors. To demonstrate our model, we applied it on a melanoma single cell dataset to identify the gene signatures that are associated with cell state and treatment response and validate the result in external bulk samples. In addition, we demonstrate the generalizability of our model to complex experimental design as well as spatially resolved single cell data from breast cancer samples.

## 2 Results

### 2.1 Interpretable Differential Abundance Signature using an ANOVA‐based Framework

**Figure 1:**
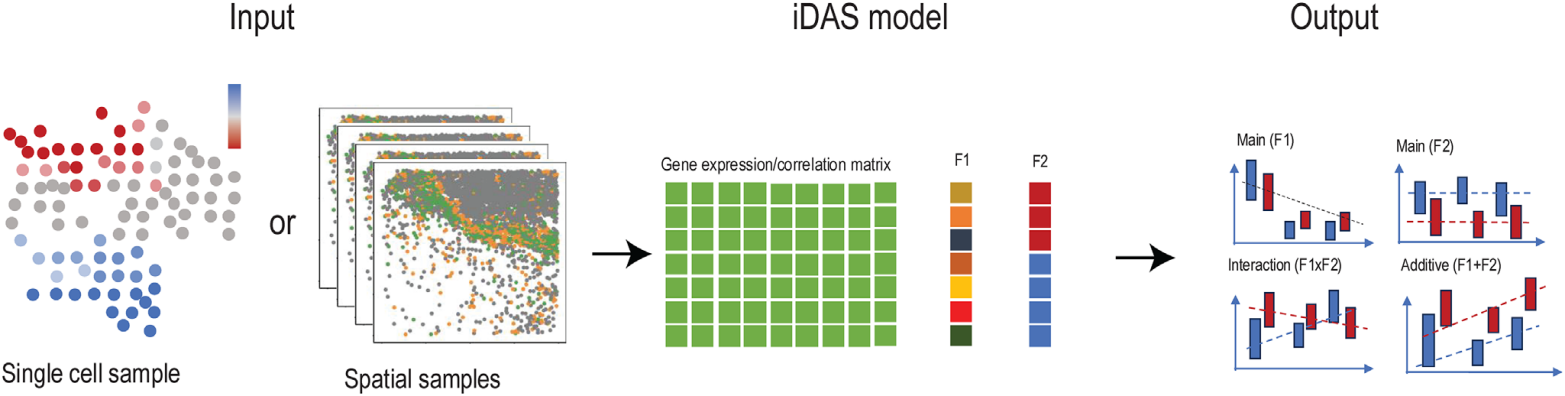
The proposed iDAS (Interpretable Differential Abundance Signature) framework identifies interpretable differential abundance signatures. iDAS can incorporate any upstream differential abundance method or spatial samples analysis results as a starting point. It creates cell type‐by‐sample pseudobulk profiles to perform ANOVA analysis, distinguishing genes significantly related to any main effect, F1 and F2. These genes are then further categorized into four groups: main effect (F1, F2), interaction effect (F1 × F2), and additive effect (F1+F2). To further interpret the gene signatures, additional differential expression analyses are performed to determine which category of effect each gene signature is most associated with.

We present a two‐stage ANOVA‐based post‐DA model approach to improve interpretation for perturbation studies, named interpretable differential abundance signature (iDAS). As illustrated in **Figure** **1**, for a single‐cell dataset with two phenotypes such as responding to treatment, we begin with the output from any differential abundance algorithm or sample based spatial gene analysis result. Examples include results from identifying binary phenotype from Milo or nearest neighbor correlation of genes in spatial samples. Next, we build on two‐way factorial experiments by jointly modeling cell‐type (or cell‐state factor, F1 in Figure 1) and treatment phenotypes factor (F2 in Figure 1). We then applied the approach similar to NANOVA,^[^ ^15^ ^]^ where we performed a series of “nested” ANOVA and used the results between the different models to classify genes into different categories relating to main and interaction effects (see Methods). These groups include the main effects for cell type (F1) and treatment phenotype (F2), the interaction effect between cell type and phenotype (F1xF2), the additive effect of cell type and phenotype (F1+F2), and a non‐significant group comprising genes not relevant to any of the main factors. This broad classification enhances the interpretability of various gene expression changes by distinguishing their specific associations with cell types and phenotypic responses. For each broad category, we perform different post‐hoc analysis to determine gene signature among specific phenotypes or cell types. These analyses are designed to suit the unique characteristics of each category.

### 2.2 iDAS Accurately Identify Cell States Markers on Melanoma Data

To demonstrate our framework, we applied iDAS to a public dataset measuring immunotherapy response in melanoma cells.^[^ ^16^ ^]^ The dataset includes 20 patients, with samples taken before and during immunotherapy treatment. We used the pre‐treatment samples of 18 patients after filtering out patient‐specific cell states not commonly present in melanoma. This resulted in 8789 cells and 14 346 genes across 9 cell states being used in the differential abundance analysis with Milo. Patient‐level phenotypes (responder and non‐responder) were used as the conditions of interest to calculate cell presence in each neighborhood (see Figure S1a, Supporting Information). In total, we identified 416 DA with *log*_2_ *FC*  ≥  5(see Figure S1b, Supporting Information). Selected DA in neighborhood graph, along with log_2_ fold change of differential abundance cells across patients, cell states, and patient phenotypes, are shown in Figure S1c–f (Supporting Information). Specially, the DA groups of responding and non‐responding cells co‐appeared in several cell states, such as mitochondrial, melanocytic, and antigen‐presenting cell states, this suggests that there may be gene signature associated with both cell state and phenotype (Figure S1e, Supporting Information).

Next, we aimed to discover the gene signatures that affect the immunotherapy phenotype and cell states by applying iDAS to the selected 4547 cells (Figure S1c, Supporting Information). Here we performed the iDAS analysis with factor 1 (F1) defined as cell state and factor 2 (F2) defined as patient‐level phenotype. Our result shows 158 genes were classified as factor 1 (cell state), 27 genes were classified as factor 2 (patient‐level phenotype), 63 genes were classified as interaction effect genes and 33 genes were classified as additive effect genes (see File S1, Supporting Information). The remaining 13 791 genes were not significantly associated with either cell state or phenotype. To highlight the interpretative advantage of iDAS, we compared it with a standard differential expression workflow using the same pseudobulk dataset. As shown in Figure S2 (Supporting Information), while traditional DE methods can model complex designs, they typically evaluate each coefficient independently without resolving overlapping contributions across factors (File S2, Supporting Information). Thus, making the cell‐type specific phenotype changes more challenging to interpret.

**Figure 2:**
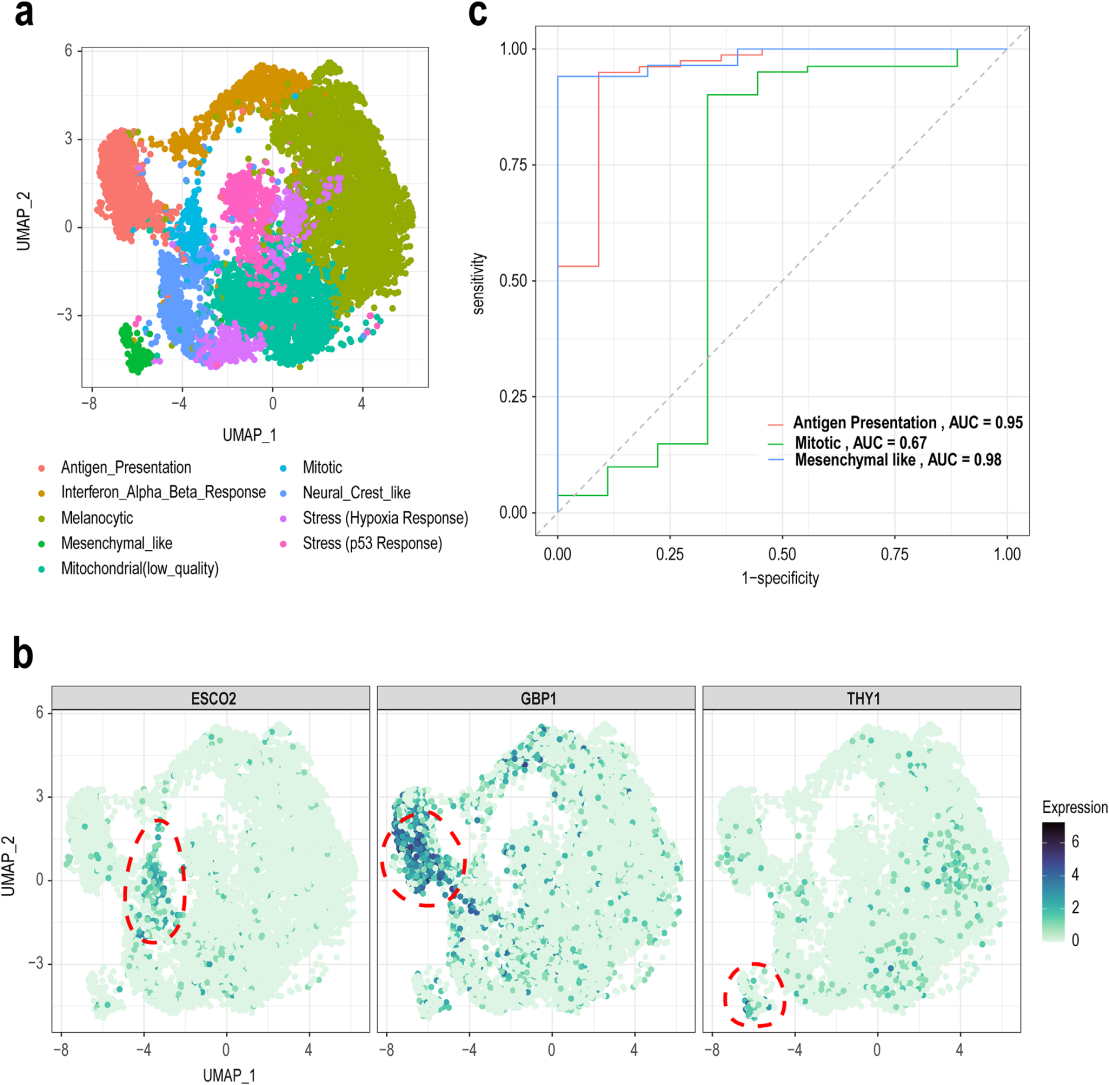
iDAS cell state associated main effect signatures. a) UMAP plot of cell state annotations in Pozniak2024 data. b) UMAP plot of single cell data, highlighting the top main effect signatures for three cell states. Gene expressions are shown for *GBP1* in antigen‐presenting cells, *ESCO2* in mitotic cells, and *THY1* in mesenchymal‐like cells. c) The ROC curves depict the performance of classification models distinguishing each cell state (Antigen‐Presentation, Mesenchymal‐like, Mitotic) from all other cell states in the dataset. The area under the curve (AUC) scores are as follows: Antigen‐Presentation (AUC = 0.97), Mesenchymal‐like (AUC = 0.86), and Mitotic (AUC = 0.87).

To assess whether the iDAS approach is able to identify known cell state markers, we examine the gene markers associated with the various cell states (F1, see **Figure** **2**a) based on differential gene expression analysis on each cell state. Figure 2b shows that these top markers are highly expressed in specific cell states as expected and align with the original study.^[^ ^16^ ^]^ For example, we identify *ESCO2* in mitotic cells (log_2_FC = 1.02; adjusted p‐value<10^−10^), *GBP1* in antigen‐presenting cell state (log_2_FC = 2.27; adjusted p‐value<10^−10^), and THY1 in mesenchymal‐like cells (log_2_FC = 0.78; adjusted p‐value<10^−10^). In parallel, we also assess the predictability of these cells using a qualitative metric and this is achieved by calculating the average area under the ROC curve (AUC) based on 10‐fold nested cross‐validation on a prediction model. The discrimination capacity of cell state‐specific gene signatures with AUC from 0.52 to 0.99 (see Figure S3, Supporting Information). Figure 2c shows strong discrimination capacity for the example cell states, and the highest cell state is antigen presentation. We also performed an analysis of hallmark gene enrichment for all cell state positive markers (log_2_FC > 0), and six cell states have markers enriched from the hallmark gene list (see Figure S4, Supporting Information). Positive markers of antigen presentation and interferon alpha/beta response cell states are both enriched in the interferon gamma and alpha response hallmark gene sets, while markers of the mitotic cell state are enriched in the E2F targets, G2M checkpoint, and mitotic spindle hallmark gene sets (see File S1, Supporting Information). These results are consistent with the original publication, demonstrating that our method can effectively identify main effect‐related signatures.

### 2.3 Application on Melanoma Data Illustrates iDAS Capacity to Identify Cell State Specific Immunotherapy Response Gene Signatures

**Figure 3:**
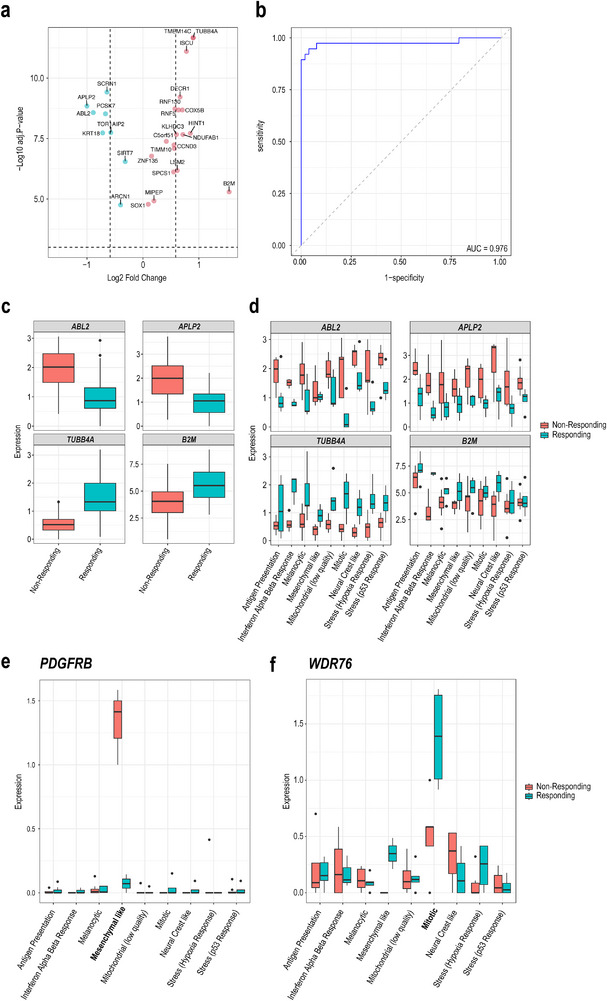
iDAS identifies the phenotype associated main effect signatures and interaction effect signatures between phenotype and cell states. a) Volcano plot illustrates the phenotype main effect signatures. The horizon dashed line indicates the significance threshold at p‐value < 0.001 (‐log_10_(p‐value) > 3). The vertical dashed lines mark the fold change thresholds at log_2_ fold change > log_2_(1.5) and log_2_ fold change < ‐log_2_(1.5). Upregulated genes in responding cell groups are colored red, and in non‐responding cell groups are colored blue. b) ROC curve evaluates the performance of a predictive model using phenotype associated main effect signatures to distinguish between responding and non‐responding samples. The area under the curve (AUC) is 0.976. c) The box plots display the expression levels of selected genes (*ABL2*, *APLP2*, *TUBB4A*, *B2M*) in responding and non‐responding samples. For *ABL2* and *APLP2*, expression is higher in non‐responding samples compared to responding samples, while for TUBB4A and B2M, expression is higher in responding samples. d) Boxplot of expression levels of the selected genes (*ABL2*, *APLP2*, *TUBB4A*, *B2M*) between the responding and non‐responding groups. Pseudobulk samples are grouped based on phenotype and cell state, with colors representing the phenotype in each category. Boxplot of e,f) are interaction effect signatures expression (*PDGFRB* and *WDR76*) across all cell states. e) *PDGFRB* is highly expressed in non‐responding groups of mesenchymal‐like cells. f) *WDR76* is highly expressed in responding groups of mitotic cells.

To identify gene signatures specific to either the responding group or the non‐responding group, we examine the genes associated with the treatment factor (F2). From the identified gene markers associated with the main effect F2, we found 11 genes significantly upregulated in the responding groups and 5 genes significantly upregulated in the non‐responding groups (see **Figure** **3**a), the other genes showed only small log_2_ fold changes. Within sample cross‐validation shows these genes are able to predict the treatment response label of pseudobulk samples with AUC of 0.976 (see Figure 3b). Figure 3c shows the expression of four example genes between the responding and non‐responding groups. *TUBB4A* is significantly upregulated in the responding group, and has been linked to melanoma‐associated pathways in a previous study,^[^ ^17^ ^]^ suggesting its potential as a treatment response marker. Another example of upregulation in the responding group is *B2M* gene. A previous study found it expressed in either tumor or stroma are associated with a positive response to immunotherapy in a metastatic melanoma patient cohort.^[^ ^18^ ^]^ We also found that *ABL2* and *APLP2* are the most significantly upregulated genes in the non‐responding cell group (see Figure 3c). This aligns with a study showing ABL1/2 drives resistance to BRAF/MEK inhibitors in melanoma^[^ ^19^ ^]^ and *APLP2* decreases HLA class I expression,^[^ ^20^ ^]^ which is linked to de‐differentiation and resistance to PD‐1 inhibition.^[^ ^21^ ^]^ Additionally, Figure 3d shows that the expression patterns of these genes in the responding and non‐responding groups are consistent across all cell states, suggesting that these genes are mainly associated with the treatment phenotype rather than cell state.

In addition to the main effect which represents overall response signature across all cell states, the power of iDAS is the ability to identify cell state specific signatures, which are formulated as gene signatures associated with the interaction effect in the iDAS framework. Figure 3e,f highlight two genes showing interaction effects between cell state and treatment phenotype. We found *PDGFRB* is highly expressed in the non‐responding group of mesenchymal‐like cells (Figure 3e; Figure S5a, Supporting Information), previous studies have shown *PDGFR* family play a significant role in brain, breast, colorectal, melanoma and lung cancers,^[^ ^22^, ^23^ ^]^ suggesting that it may be potential marker for treatment response. Another example is the gene *WDR76* which is observed to be highly expressed in the responding group of mitotic cells (Figure 3f; Figure S5b, Supporting Information). Current literatures have shown that *WDR76* function as tumor suppressor in colorectal, bladder, colon and liver cancer,^[^ ^24^, ^25^ ^]^ showing that it may also be potential marker for treatment response in melanoma.

### 2.4 Identification of Overall and Cell‐Specific Immunotherapy Response Gene Signatures in Bulk Expression Data from Independent Cohorts

**Figure 4:**
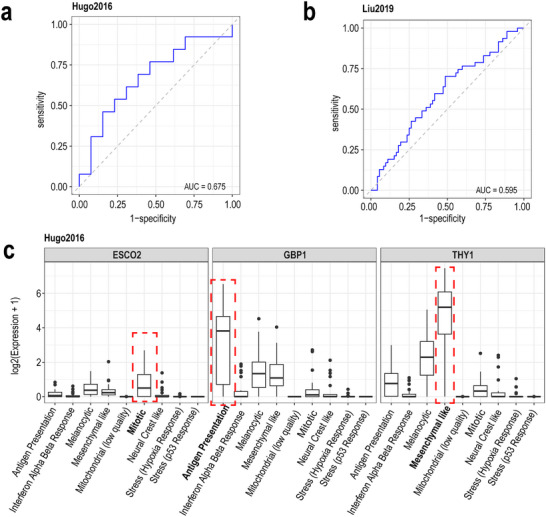
Main effect signature validation using independent cohort from bulk data (Hugo2016 and Liu2019). a,b) AUC plots shows 10‐fold cross‐validation to predict bulk sample responses using gene signatures derived from iDAS, analyzed via generalized linear models. c) shows cell state main effect signatures are transferable to external bulk data. The box plot shows the expression of genes *ESCO2*, *GBP1* and *THY1*, mapped onto cell state specific deconvolutions of external bulk RNA dataset. Consistent with the observations in Figure 2b, these genes exhibit high expression in particular cell states, underscoring their role as specific markers across different datasets.

After demonstrating that the signatures are meaningful within the single cell dataset, we aimed to see the translational potential of these signatures by examining the fold‐change of these markers in two other melanoma bulk RNA‐seq data, Liu2019^[^ ^26^ ^]^ and Hugo2017 dataset.^[^ ^27^ ^]^ In general, we note that the expression of these markers are not always high in the bulk samples. Thus, to further evaluate the predictive ability of phenotype‐associated main effect signatures, we only selected genes with log_2_FC greater than log_2_(1.3) as features. Here, we built a generalized linear model to predict the phenotype of bulk samples. The AUC of 10‐fold nested cross‐validation is 0.675 from the Hugo2016 dataset (**Figure** **4**a) and 0.595 from Liu2019 dataset (Figure 4b). This result suggests that the signatures discovered in the single‐cell context are also predictive markers in a bulk RNA sequencing setting, reinforcing their potential utility as robust biomarkers.

To assess the cell state markers identified from the single cell data in the bulk we examine the deconvoluted bulk RNA‐seq data. This analysis confirmed that the marker genes, highly expressed in specific cell states in single‐cell data, retain similar expression profiles in the deconvoluted bulk RNA data. Consequently, these markers are validated as conserved cell‐ markers across various datasets, as illustrated in Figure 4c and Figure S6 (Supporting Information). We next examine the expression profile of the interaction effect signatures on the bulk deconvoluted dataset. Specifically, we examined the expression levels of *WDR76* and *PDGFRB* in the context of patient response to immunotherapy. Our results show that *WDR76* is slightly more highly expressed in the responding group than in the non‐responding group in the mitotic cell state in the Hugo2016 dataset (see Figure S7a, Supporting Information). Its differential expression is more pronounced in the Liu2019 dataset (see Figure S7c, Supporting Information). Although Figure S7b (Supporting Information) shows a significant differential expression of *PDGFRB* in mesenchymal‐like cells in the Hugo2017 dataset, the Liu2019 data does not support it as a non‐responsive marker gene of mesenchymal‐like cells (see Figure S7d, Supporting Information). Taken together, these results suggest that some of the cell state specific markers (interaction effect signatures) identified in the single‐cell data are also reflected in the bulk data.

### 2.5 iDAS can Incorporate any Upstream Differential Abundance Method

In the above analysis, we primarily focus on identifying interpretable gene signatures using a differential abundance method called Milo. We then apply the same iDAS analysis with two other well‐established differential abundance detection methods, DA‐seq and Scissor. Using DA‐seq, we identified 2437 responding cells and 4460 non‐responding cells, whereas Scissor identified 3006 responding cells and 5783 non‐responding cells (see Figure S8, Supporting Information). We then applied iDAS to the selected differentially abundant cells. iDAS identified 155 genes associated with cell state, 36 with patient‐level phenotype, 59 with the interaction effect, and 35 with the additive effect for DA‐seq. For Scissor, iDAS identified 217 genes associated with cell state, 6 with patient‐level phenotype, 19 with the interaction effect, and 44 with the additive effect.

However, the patient‐level phenotype‐associated genes identified by iDAS varied significantly depending on the upstream differential abundance (DA) detection method—Milo, DA‐seq, or Scissor (see Figure S9, Supporting Information). Only two genes were shared across all three DA methods. In contrast, the majority of cell state‐associated genes were consistently identified across the three methods. For the interaction effect category, only three genes were shared, while in the additive effect category, only four genes overlapped. These differences suggest that the variability arises primarily from the choice of DA detection method rather than from iDAS itself. Unlike Milo, which quantifies the number of cells from each sample within k‐nearest neighbors and applies a negative binomial generalized linear model (NB‐GLM) to test differential abundance (DA) in each local graph, DA‐seq uses a logistic regression model to predict DA scores for individual cells under two distinct conditions.^[^ ^28^ ^]^ While Milo and DA‐seq rely solely on single‐cell data to identify differential abundance, Scissor integrates bulk data as a reference, mapping bulk sample phenotypes to single cells by optimizing a regression model based on the correlation matrix between bulk and single‐cell data. These differences could lead to variations in detecting composition and expression shifts in the data.

### 2.6 Extensibility of iDAS to Account for Longitudinal Studies and to Spatially Resolved Omics Data

**Figure 5:**
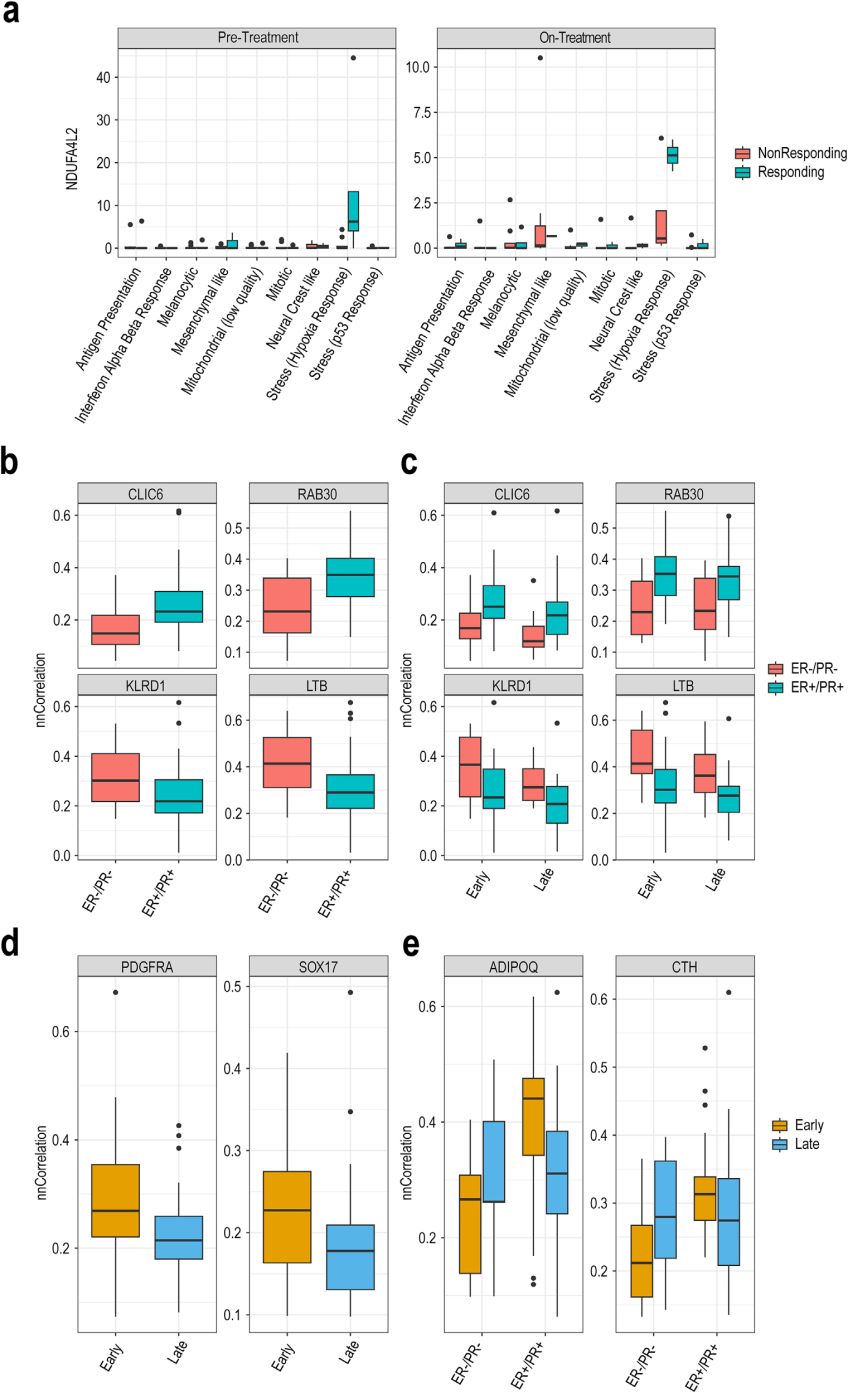
Application of iDAS to longitudinal and spatial results. a) Boxplot shows the expression levels of an interaction effect (F2F3) signature across various cell states in both pre‐treatment and on‐treatment conditions. *NDUFAL2* is highly expressed in stress (hypoxia response) specific cell state among responding groups. b) Boxplots showing nearest neighbor correlation of *CLIC6* and *RAB30*, indicating their specificity to ER/PR status. These genes demonstrate a strong correlation with ER+/PR+ group than ER‐/PR‐ group. c) Boxplots showing that the nearest neighbor correlation of the same two genes in panel (b) is consistent across both early and late stages, indicating that their expression is not dependent on the stage of disease progression. d) Boxplots illustrating the nnCorrelation of two stage‐specific genes, showing differential expression between early and late AJCC pathological stage of the disease. e) Boxplots depicting interaction effect between ER/PR status and AJCC pathological stage for *ADIPOQ* and *CTH*, with varying expression levels depending on both factors.

The flexibility of iDAS model can be illustrated by its ability to handle multi‐factor longitudinal data. For the same melanoma dataset, we can also model by accounting for pre‐ and on‐treatment status for a given individual. Here, we define pre‐ and on‐treatment as factor F1, treatment phenotype status (responding/non‐responding) as factor F2, and cell state as factor F3. This three‐way model allows us not only to identify the main effects associated with F1, F2, and F3, but also to detect two‐way interaction effects, such as F1F2, F1F3, and F2F3. By running iDAS using a three‐way random effects model, we identified a total of 350 genes that may be associated with one of the factors. We found 19 interaction effect signatures, including 18 three‐way effects and 1 two‐way effect, along with 331 main effect signatures (see File S1, Supporting Information). The only two‐way interaction effect gene is *NDUFA4L2*, which is a marker for the stress (hypoxia response) cell state in responding group, regardless of pre‐ or on‐treatment status (see **Figure** **5**a). For the main effect signatures, one gene is associated with factor F1, 321 genes are associated with factor F3, and 9 genes are classified under additive effect categories. We found that the cell state‐associated genes, specifically the positive markers of seven cell states (see Figure S10, Supporting Information), are enriched in the hallmark gene lists, with most of them consistent with the original publication (see File S1, Supporting Information).

Beyond the analysis of single‐cell samples, iDAS framework can also be applied to examine spatially resolved transcriptomics data. For example, we used GHIST‐predicted TCGA breast cancer spatial transcriptomics data to calculate the nearest neighbor correlation for each gene across all cell pairs (see Methods). We then modeled the spatial metric as an input to investigate the spatial relationships of gene expression in relation to ER/PR status and AJCC pathological stage. As shown in Figure 5b,c, we found that the correlation of expression for *CLIC6* and *RAB30* in neighboring cells is higher in the ER+/PR+ group, while the correlation of expression for *KLRD1* and *LTB* is higher in the ER‐/PR‐ group, regardless of whether the tumor is detected in the early or late stage. To explore tumor stage‐associated genes, we discovered that the correlation of expression for *PDGFRA* and *SOX17* in neighboring cells is higher in early‐stage tumor patients but not in late‐stage patients (see Figure 5d). Besides, we identified two genes, *ADIPOQ* and *CTH*, that exhibit a mixed pattern across tumor stages and ER/PR status. These genes are highly correlated in neighboring cells of ER+/PR+ patients in the early‐stage group but not in ER‐/PR‐ patients (see Figure 5e).

## 3 Discussion

In this study, we introduced iDAS, an ANOVA‐based post‐DA framework designed to interpret gene signatures from differential abundance analysis. The novelty of iDAS lies in its ability to systematically dissect main and interaction effects across multiple experimental factors. This analytical perspective is rarely addressed in current differential abundance workflows. Using a melanoma dataset, we demonstrated that iDAS effectively identifies both overall responding signatures (main effect) and cell‐state specific responding signatures (interaction effect). We identified cell state‐specific signatures that have the power to distinguish between different cell states. We found upregulated and downregulated genes in both responding and non‐responding groups, indicating potential markers for treatment response and non‐response. Signatures were further validated on bulk datasets, demonstrating the transferability of the signature selected by iDAS. Lastly, we demonstrate the flexibility of the model by applying iDAS to more complex study designs, including longitudinal data with pre‐ and on‐treatment measurements from the same individual using three‐way analysis, and in‐silico spatially resolved transcriptomics data.

One of the strengths of iDAS's results is that the gene signatures found in a smaller single cell dataset is transferable to larger bulk RNA‐sequencing datasets demonstrating its translational capacity. However, the validation strategies associated with both overall and cell‐type specific markers are imperfect as they may depend on the difference between platforms as well as the performance of deconvolution approaches. For example, while our study shows very similar expression trends between single cell and bulk datasets, we notice that some genes of interest found in single cell are not “highly expressed” bulk. Hence, validation across platforms should be conditioned on “expressed genes”. In addition, the actual fold‐change estimate in deconvoluted bulk datasets are much lower than single cell data. Possible explanations include masking of signals from rare cell states,^[^ ^29^ ^]^ cell states in the reference differing from the bulk data(Avila^[^ ^30^, ^31^ ^]^), and lack of raw counts as input from bulk samples. Thus, improving deconvolution methods will increase the reliability of translating single‐cell insights to bulk RNA‐seq data, enabling more accurate for validation. It is important to note that in practice deconvolution is not required for cell state implementation as there exist other biotechnological platform strategies to select appropriate cell state.

The iDAS framework offers much greater flexibility in handling complex experimental designs than pairwise DE analyses. First of all, in contrast to a series of pairwise DE analyses, iDAS can perform multiple group comparisons simultaneously. Furthermore, the proposed framework is flexible enough to handle longitudinal studies where iDAS can easily include random effects in the models when the data includes repeated measurements from the same patients such as pre‐ and on‐treatment information. This addition provides a more accurate understanding of each effect in the model between different experimental factors.

In our external bulk validation, we focus on the validation of the markers and not necessarily the validation of the actual “prediction model”. That is, we train a new model with the signature expression profile using the bulk data to predict the treatment phenotype on the same data. The ability to integrate models between single‐cell and bulk data is a topic of interest in data harmonization within the single‐cell community. There are methods in bulk data, such as log‐ratio approaches, that enable transfer learning across cohorts.^[^ ^32^ ^]^ Future work will be to develop single cell extension to these approaches and integrate it into the iDAS framework.

iDAS can incorporate any upstream differential abundance method, though the identified gene signatures may vary across different DA methods. We found different DA methods can produce different cell state results due to their different design approaches. To improve the results of the iDAS gene signature, further research should be conducted to develop an intermediate step to harmonize cell states generated by different methods and to obtain stable gene signatures across DA methods. One approach to improve the stability of cell state labels is to apply AdaSampling.^[^ ^33^ ^]^ It can iteratively reclassify cell state labels to reduce noise and correct misclassification, leading to more consistent and reliable cell states across different DA methods.

As an ANOVA‐based framework, whether datasets meet the assumptions for performing ANOVA testing is a main concern for researchers. In our workflow, we create pseudobulk per cell type per sample instead of aggregating all cells within a sample. This preserves major differences among cell types while reducing technical noise. Since iDAS aims to identify genes linked to treatment effects while minimizing cell type or state influences, this approach aligns with our objective and enhances robustness. Besides, creating pseudobulk helps reduce overdispersion and brings the data closer to a normal distribution, which is an important assumption for performing ANOVA. While the homogeneity of variance (homoscedasticity) may not always hold in two‐factor or three‐factor analyses, the iDAS model allows users to select the top percentage of genes instead of relying on a strict p‐value cutoff. In many cases, selecting the top 5% or 10% of genes is more useful for research purposes than sticking to a fixed threshold. A nonparametric approach could also avoid the assumptions of ANOVA. However, with more than 15 000 genes, running such tests would be extremely time‐consuming. Further research is needed to explore more efficient solutions in this area.

While iDAS provides a flexible and interpretable framework for identifying gene signatures associated with mean and interaction effects across multiple experimental factors, its scalability is limited. iDAS only supports up to three factors. As the number of factors increases, the number of potential interaction terms grows combinatorially, leading to challenges in statistical power, increased model complexity, and diminished interpretability. Moreover, iDAS operates on pseudobulk data aggregated by cell types, which limits its sensitivity to detect subtle within‐cell‐type heterogeneity or continuous cellular trajectories. These features are better captured by single‐cell–level models.

In summary, to enhance the interpretability of differential abundance signatures, we introduce an ANOVA‐based framework, iDAS, that is able to identify the signature from multiple conditions at once. Our findings confirm that iDAS framework can effectively identify robust cell‐state specific gene signatures and the framework is flexible to handle various complex study designed including multi‐factor longitudinal and spatially resolved data.

## 4 Experimental Section

### Dataset and Processing

In this study, it was used three public datasets of melanoma immunotherapy patients to demonstrate the iDAS framework: one single cell RNA‐seq dataset and two bulk RNA‐seq datasets.

#### Pozniak2024

This single cell RNA‐seq data is from a longitudinal profiling study of metastatic melanoma under immune checkpoint blockade (ICB).^[^ ^16^ ^]^ Biopsies were taken from cutaneous, subcutaneous, or lymph node metastases before treatment (BT) and early on‐treatment (OT; before the second ICB infusion) and processed for scRNA‐seq using the 10x Genomics Chromium platform. For two‐way iDAS analysis, it was used the pre‐treatment data, filtering out cell states named Patient‐specific‐A and Patient‐specific‐B and only including protein coding genes. This resulted in a dataset of 18 patients with 8789 cells and 14 346 genes for the differential abundance analysis to identify significant DA groups. For the three‐way iDAS analysis, we used the pre‐ and on‐treatment data together, which includes 13 789 cells and 13 887 genes. The single cell dataset was mapped to the GRCh38 genome reference.

#### Liu2019

This bulk RNA‐seq data comes from a cohort of patients with advanced melanoma who received PD1 blockade as palliative treatment.^[^ ^26^ ^]^ FFPE tissues were obtained before PD‐1 blockade. Patients were classified into five clinical statuses in the original study: Complete Response (CR), Partial Response (PR), Minor Response (MR), Progressive Disease (PD), and Stable Disease (SD). For this analysis, CR and PR as responders (n = 47) and PD and SD as non‐responders (n = 72) resulting in a dataset of 119 individuals was selected. The data was aligned using STAR with GRCh38 reference and quantified with RSEM to yield gene‐level expression in transcripts per million (TPM). It was directly used the TPM matrix for our analysis.

#### Hugo2017

This bulk RNA‐seq data comes from a cohort of patients with metastatic melanoma who received either pembrolizumab or nivolumab as their anti‐PD‐1 therapy.^[^ ^27^ ^]^ Melanoma specimens before treatment with sufficient RNA quality were analyzed by RNA‐Seq. Paired‐end transcriptome reads were mapped to the UCSC hg19 reference genome using Tophat2. Normalized expression levels of genes were reported in FPKM values. It was directly used the FPKM matrix of 26 patients for this analysis.

### iDAS Framework

iDAS involves two main components, the first component uses a series of nested ANOVA‐statistics to classify genes into different groups, and the second component performs relevant differential expression analysis for signature identification and interpretation. Each step was described in the following subsections.

[A] ANOVA‐based test to classify genes

[A1] Two‐way fix effect model

The development of the ANOVA‐based tests in our framework was inspired by the nonparametric ANOVA (NANOVA) method,^[^ ^15^ ^]^ which was originally proposed for classifying genes based on their factor effects in microarray data. In this approach, gene expression was modeled as the response variable and the cell type or experiment factors as explanatory variables. Specifically, in two‐way factorial experiments, it could summarize gene expression by one of the following ANOVA models.

(1) $$\mathrm{Model}\;1\;-\;\;y_{ijm}=\mu +\alpha_{i}+\beta_{j}+{\left(\alpha \beta \right)}_{ij}+\varepsilon_{ijm}$$

(2) $$\mathrm{Model}\;2\;-\;\;y_{ijm}=\mu +\alpha_{i}+\beta_{j}+\varepsilon_{ijm}$$

(3) $$\mathrm{Model}\;3\;-\;\;y_{ijm}=\mu +\alpha_{i}+\varepsilon_{ijm}$$

(4) $$\mathrm{Model}\;4\;-\;\;y_{ijm}=\mu +\beta_{j}+\varepsilon_{ijm}$$

(5) $$\mathrm{Model}\;5\;-\;\;y_{ijm}=\mu +\varepsilon_{ijm}$$

In these models, α_*i*_ with {i:1,…,I}, and β_*j*_ with {j:1,…,J} denote the two factors of interest at levels *i* and *j*, respectively, and *y*_{*g*,*ijm*}_represents the expression of gene *g* under the condition defined by (α_*i*_,β_*j*_). Here, *m* with $m=1,\;\text{…}.,\;m_{ij}$ is a subscript for replicates. The full model (Model 1) includes 𝜇 representing the baseline gene expression and (αβ)_*ij*_as the interaction term, indicating that genes were influenced by both factors and the effect of one factor depends on the level of the other. The “additive” model (Model 2) shows that genes are affected by both factors independently, without any interaction effect. “Single‐factor” models (Models 3 and 4) illustrate genes affected by only one factor, either α_*i*_ or β_*j*_. Lastly, the “null” model (Model 5) indicates that gene expression was not influenced by either factor.

Next, iDAS classified genes into five groups (*C*_*F*1_, *C*_*F*2_, *C*_*F*1 × *F*2_,  *C*_*F*1 + *F*2_,  *C*_*non* − *sig*_), each corresponding to one of the models described earlier (see Figure S11b, Supporting Information). This classification was based on a series of ANOVA tests as detailed below.

1) *No effect test*: In this step, a test was conducted to determine whether the gene expression is affected by any factor, corresponding to the null hypothesis $H0:\;\;\alpha_{i}=\beta_{j}={\left(\alpha \beta \right)}_{ij}=0;\;i=1,\text{…},I,\;j=1,\;\text{…},\;J$(Model 5) versus alternative hypothesis $H0:\;\forall \;\alpha_{i}\neq 0,\;\beta_{j}\neq 0,\;{\left(\alpha \beta \right)}_{ij}\neq 0;\;i=1,\text{…},I,\;j=1,\;\text{…},\;J$ (Model 1). The p‐values were then adjusted using the Benjamini & Hochberg method to control the false discovery rate. The significance level of the p‐value was set using two methods: either by ordering the p‐values and defining the top 2% as the significance level, or by using a predefined significance level, such as 0.05 or 0.01. Genes with p‐values larger than this significance level are considered part of the non‐significant group (*C*_*non* − *sig*_). The significant genes (*C_sig_*) were further classified into four groups (*C*_*F*1_, *C*_*F*2_, *C*_*F*1 × *F*2_,  *C*_*F*1 + *F*2_).
2) *Interaction effect test*: *C_sig_* was then further classified into interaction effect and non‐interaction effect groups. The null hypothesis is $H0:\;\;{\left(\alpha \beta \right)}_{ij}=0;\;i=1,\text{…},I,\;j=1,\;\text{…},\;J$(Model 2), indicating no interaction effects between factor 1 and factor 2. The alternative hypothesis is $H1:\;{\left(\alpha \beta \right)}_{ij}\neq 0$; ∀i=1,…,I,j=1,…,J, which corresponds to Model 1. Follow the same idea from “No effect test”, the set of gene signatures with significant interaction effects is defined as having p‐values less than significance level and is denoted as *C*_*F*1 × *F*2_.
3) *Main effect test*: To test the main effect of α (F1), the null hypothesis is $H0:\;\alpha_{i}=0;\;i=1,\;\text{…},\;I$, indicating no effect of F1 on the gene signatures, which corresponds to Model 4. The alternative hypothesis is $H1:\;\alpha_{i}\neq 0;\;\forall \;i=1,\;\text{…}\;I$, corresponding to Model 2.

Similarly, to test the main effect of β (F2), the null hypothesis is $H0:\;\beta_{j}=0;\;j:\;1,\;\text{…},\;J$, indicating the set of gene signatures with no F2 effect, which corresponds to Model 3. The alternative is still Model 2, $H1:\beta_{j}\neq 0;\;j=1,\;\text{…},\;J$.
The two tests described above were applied to the genes set *C_sig_* ∖ *C*_*F*1 × *F*2_ to identify genes having α (F1) and β(F2) effect, respectively. Denote these two sets as *C*_α_ and *C*_β_, then it could get *C*_*F*1 _ = *C*_α_ ∖ {*C*_α_ ∩ *C*_β_} and *C*_*F*2_ = *C*_β_ ∖ {*C*_α_ ∩ *C*_β_}.

4) *Additive effect*: After performing the three tests described above, the remaining genes in *C_sig_* are classified into *C*_*F*1 + *F*2_, representing the additive effect group. This set was defined as *C*_*sig*_∖ {*C*_*F*1 × *F*2_ ∩ *C*_*F*1_ ∩ *C*_*F*2_}.

[A2] Three‐way random effect model

In this section,a three‐way ANOVA classification model with a random effect was constructed. Specifically, in three‐way factorial experiments, gene expression can be summarized using one of the following ANOVA models..

(6) $$\begin{array}{ccc} \mathrm{Model}\;1\; & & -\;\;y_{ijknm}=\mu +\alpha_{i}+\beta_{j}+\gamma_{k}+{\left(\alpha \beta \right)}_{ij}+{\left(\beta \gamma \right)}_{jk}+{\left(\alpha \gamma \right)}_{ik}+{\left(\alpha \beta \gamma \right)}_{ijk}\\ & & +u_{n}+\varepsilon_{ijknm} \end{array}$$

(7) $$\begin{array}{ccc} \mathrm{Model}\;2\; & & -\;\;y_{ijknm}=\mu +\alpha_{i}+\beta_{j}+\gamma_{k}+{\left(\alpha \beta \right)}_{ij}+{\left(\beta \gamma \right)}_{jk}+{\left(\alpha \gamma \right)}_{ik}\\ & & +u_{n}+\varepsilon_{ijknm} \end{array}$$

(8) $$\mathrm{Model}\;3\;-\;\;y_{ijknm}=\mu +\alpha_{i}+\beta_{j}+\gamma_{k}+{\left(\beta \gamma \right)}_{jk}+{\left(\alpha \gamma \right)}_{ik}+u_{n}+\varepsilon_{ijknm}$$

(9) $$\mathrm{Model}\;4\;-\;\;y_{ijknm}=\mu +\alpha_{i}+\beta_{j}+\gamma_{k}+{\left(\alpha \beta \right)}_{ij}+{\left(\alpha \gamma \right)}_{ik}+u_{n}+\varepsilon_{ijknm}$$

(10) $$\mathrm{Model}\;5\;-\;\;y_{ijknm}=\mu +\alpha_{i}+\beta_{j}+\gamma_{k}+{\left(\alpha \beta \right)}_{ij}+{\left(\beta \gamma \right)}_{jk}+u_{n}+\varepsilon_{ijknm}$$

(11) $$\mathrm{Model}\;6\;-\;\;y_{ijknm}=\mu +\alpha_{i}+\beta_{j}+\gamma_{k}+u_{n}+\varepsilon_{ijknm}$$

(12) $$\mathrm{Model}\;7\;-\;\;y_{ijknm}=\mu +\alpha_{i}+u_{n}+\varepsilon_{ijknm}$$

(13) $$\mathrm{Model}\;8\;-\;\;y_{ijknm}=\mu +\beta_{j}+u_{n}+\varepsilon_{ijknm}$$

(14) $$\mathrm{Model}\;9\;-\;\;y_{ijknm}=\mu +\gamma_{k}+u_{n}+\varepsilon_{ijknm}$$

(15) $$\mathrm{Model}\;10\;-\;\;y_{ijknm}=\mu +u_{n}+\varepsilon_{ijknm}$$

In these models, α_*i*_ with {i:1,…,I}, β_*j*_ with {j:1,…,J} and γ_*k*_ with {k:1,…,K} denote the three factors of interest at levels *i*, *j* and *k*, respectively, and *y*_{*g*,*ijknm*}_represents the expression of gene *g* under the condition defined by (α_*i*_,β_*j*_,γ_*k*_) . Here, *n* with $n=1,\;\text{…}.,\;N_{ijk}$ is a subscript for random effect factor, *m* with $m=1,\text{…},M_{ijk}$ is a subscript for replicates. The baseline gene expression is denoted as 𝜇. (αβγ)_*ijk*_is the interaction term, indicating that genes were influenced by three factors and the effect of one factor depends on the level of the others. (αβ)_*ij*_, (βγ)_*jk*_and (αγ)_*ik*_ are the interaction term of two factors. Model 1 was the full model, including all three factors with two‐way and three‐way interaction effects, and a random effect *u_n_*. Model 2 only includes two‐way interaction effects. Models 3, 4, and 5 include only two‐way interaction effects, but each model excludes one of the two‐way interaction effects. Model 6 illustrates that genes were affected by three factors independently, without any interaction effect. Models 7,8, and 9 were single factor models, showing that genes were affected by only one factor. Lastly, Model 10 indicates that gene expression was not influenced by any factor.

Next, genes were classified into ten groups

(*C*_*F*1_, *C*_*F*2_, *C*_*F*3_, *C*_*F*1 × *F*2_,  *C*_*F*2 × *F*3_,  *C*_*F*1 × *F*3_, *C*_*two*−*way* *combine*_,  *C*_*additive*_,*C*_*F*1 × *F*2 × *F*3_,  *C*_*non*−*sig*_),

 each corresponding to one of the models described earlier (see Figure S11c, Supporting Information). This classification is based on a series of ANOVA tests as detailed below. This classification is based on a series of ANOVA tests as detailed below.

1) *No effect test*: In this step,a test was conducted to determine whether the gene expression is affected by any factor, corresponding to the null hypothesis $H0:\;\;\alpha_{i}=\beta_{j}=\gamma_{k}={\left(\alpha \beta \right)}_{ij}={\left(\beta \gamma \right)}_{jk}={\left(\alpha \gamma \right)}_{ik}={\left(\alpha \beta \gamma \right)}_{ijk}=0;\;i=1,\text{…},I,\;j=1,\;\text{…},\;J,\;k=1,\text{…},K$ (Model 10) versus alternative hypothesis $H0:\;\forall \;\alpha_{i}\neq 0,\;\beta_{j}\neq 0,\gamma_{k}\neq 0,\;{\left(\alpha \beta \right)}_{ij}\neq 0,{\left(\beta \gamma \right)}_{jk}\neq 0,{\left(\alpha \gamma \right)}_{ik}\neq 0,{\left(\alpha \beta \gamma \right)}_{ijk}\neq 0;\;i=1,\text{…},I,\;j=1,\;\text{…},\;J,\;k=1,\text{…},K$ (Model 1). The p‐values were then adjusted using the Benjamini & Hochberg method to control the false discovery rate. The significance level of the p‐value was set using two methods: either by ordering the p‐values and defining the top 2% as the significance level, or by using a predefined significance level, such as 0.05 or 0.01. Genes with p‐values larger than this significance level were considered part of the non‐significant group (*C*_*non* − *sig*_). The significant genes (*C_sig_*) are further classified into other groups.
2) *Interaction effect test*: *C_sig_* was then further classified into interaction effect and non‐interaction effect groups. The null hypothesis, $H0:\;\;{\left(\alpha \beta \right)}_{ij}=0,\;\;{\left(\beta \gamma \right)}_{jk}=0,\;\;{\left(\alpha \gamma \right)}_{ik}=0,\;\;{\left(\alpha \beta \gamma \right)}_{ijk}=0;\;i=1,\text{…},I,\;j=1,\;\text{…},\;J,\;k=1,\text{…},K$(Model 6), indicating no interaction effects among factors. The alternative hypothesis is $H1:{\left(\alpha \beta \right)}_{ij}\neq 0,\;{\left(\beta \gamma \right)}_{jk}\neq 0,{\left(\alpha \gamma \right)}_{ik}\neq 0,{\left(\alpha \beta \gamma \right)}_{ijk}\neq 0;\forall \;i=1,\text{…},I,\;j=1,\;\text{…},\;J,\;k=1,\text{…},K$, which corresponds to Model 1. The set of gene signatures with significant interaction effects is denoted as *C_Int_*, while set of gene signatures without significant interaction effects is denoted as *C_NotInt_*.
3) *Main effect test*: To test the main effect of α (F1), our null hypothesis is $H0:\;\alpha_{i}\neq 0,\;\;\beta_{j}=0,\;\gamma_{k}=0;\;i=1,\;\text{…},\;I,\;j=1,\;\text{…},\;J,\;k=1,\text{…},K$, indicating no effect of β(F2) and γ(F3) on the gene signatures, which corresponds to Model 7. The alternative hypothesis is $H1:\;\alpha_{i}\neq 0,\;\beta_{j}\neq 0,\gamma_{k}\neq 0;\;\forall \;i=1,\;\text{…},\;I,\;j=1,\;\text{…},\;J,\;k=1,\text{…},K$, corresponding to Model 6.

Similarly, to test the main effect of β (F2), the null hypothesis is $H0:\;\alpha_{i}=0,\;\beta_{j}\neq 0,\;\gamma_{k}=0;\;i=1,\;\text{…},\;I,\;j=1,\;\text{…},\;J,\;k=1,\text{…},K$, indicating the set of gene signatures with only F2 effect, which corresponds to Model 8. And the alternative is still Model 6, $H1:\;\alpha_{i}\neq 0,\;\beta_{j}\neq 0,\gamma_{k}\neq 0;\;\forall \;i=1,\;\text{…},\;I,\;j=1,\;\text{…},\;J,\;k=1,\text{…},K$.
Lastly, to test the main effect of γ (F3), the null hypothesis is $H0:\;\alpha_{i}=0,\;\;\beta_{j}=0,\gamma_{k}\neq 0;\;i=1,\;\text{…},\;I,\;j=1,\;\text{…},\;J,\;k=1,\text{…},K$, indicating the set of gene signatures with only F3 effect, which corresponds to Model 9. And the alternative is still Model 6, $H1:\;\alpha_{i}\neq 0,\;\beta_{j}\neq 0,\gamma_{k}\neq 0;\;\forall \;i=1,\;\text{…},\;I,\;j=1,\;\text{…},\;J,\;k=1,\text{…},K$.
The three tests described above were applied to the genes set *C_NotInt_* to identify genes having α (F1), β(F2) and γ (F3) effect, respectively. *C*_*F*1 _ is the gene set that fails the main effect test for F1 but passes the main effect tests for F2 and F3. Conversely, *C*_*F*2 _ fails the main effect test for F2 but passes the main effect tests for F1 and F3. And *C*_*F*3 _fails the main effect test for F3 but passes the main effect tests for F1 and F2.

4) *Additive effect*: After performing the three tests described in main effect test, the remaining genes in *C_NotInt_* are classified into *C*_***additive***_, representing the additive effect group, which includes F1+F2, F1+F3, F2+F3 and F1+F2+F3.
5) *Three‐way interaction effect test*: The interaction gene set *C_Int_* was further classified into groups based on three‐way interaction effects and two‐way interaction effects. the null hypothesis is $H0:\;{\left(\alpha \beta \gamma \right)}_{ijk}=0;\;i=1,\;\text{…},\;I,\;j=1,\;\text{…},\;J,\;k=1,\text{…},K$, indicating there is no three‐way intersection effect, which corresponds to Model 2. And the alternative is $H1:\;{\left(\alpha \beta \gamma \right)}_{ijk}\neq 0;\;\forall \;i=1,\;\text{…},\;I,\;j=1,\;\text{…},\;J,\;k=1,\text{…},K$, which corresponds to Model 1. With this test, *C_Int_* can be partitioned into *C*_*F*1 × *F*2 × *F*3_ when p‐value less than the significance level, and *C*_*two* − *way*_ when p value is greater than cutoff value.
6) *Two‐way interaction effect test*: To further interpret the two‐way interaction effect genes, they were classified into four groups: F1F2, F1F3, F2F3, and a general two‐way combination group.

To test the two‐way interaction effect of (αβ) (F1F2), the null hypothesis is $H0:\;\;{\left(\alpha \beta \right)}_{ij}=0;\;i=1,\;\text{…},\;I,\;j=1,\;\text{…},\;J$, indicating no effect of (αβ) (F1F2) on the gene signatures, which corresponds to Model 3. The alternative hypothesis is $H1:\;{\left(\alpha \beta \right)}_{ij}\neq 0;\forall \;i=1,\;\text{…},\;I,\;j=1,\;\text{…},\;J$, corresponding to Model 2.
Similarly, to test the two‐way interaction effect of (βγ) (F2F3), the null hypothesis is $H0:\;\;{\left(\beta \gamma \right)}_{jk}=0;\;j=1,\;\text{…},\;J,\;k=1,\text{…},K$, indicating no effect of (βγ) (F2F3) on the gene signatures, which corresponds to Model 4. The alternative hypothesis is $H1:\;{\left(\beta \gamma \right)}_{jk}\neq 0;\forall \;j=1,\;\text{…},\;J,\;k=1,\text{…},K$, corresponding to Model 2.
Lastly, to test the two‐way interaction effect of (αγ) (F1F3), the null hypothesis is $H0:\;\;{\left(\alpha \gamma \right)}_{ik}=0;i=1,\;\text{…},\;I,\;k=1,\text{…},K$, indicating no effect of (αγ) (F1F3) on the gene signatures, which corresponds to Model 5. The alternative hypothesis is $H1:\;{\left(\alpha \gamma \right)}_{ik}\neq 0;\forall \;i=1,\;\text{…},\;I,\;k=1,\text{…},K$, corresponding to Model 2.
The tests described above were applied tothe genes set *C*_*two* − *way*_ to identify genes having (αβ) (F1F2), (βγ) (F2F3) and (αγ) (F1F3) interaction effect, respectively. *C*_*F*1 × *F*2_ is the gene set that passes the two‐way interaction effect test for F1F2 but fails the main effect tests for F2F3 and F1F3. Conversely, *C*_*F*2 × *F*3 _ passes the main effect test for F2F3 but fails the main effect tests for F1F2 and F1F3. And *C*_*F*1 × *F*3 _ passes the main effect test for F1F3 but fails the main effect tests for F1F2 and F2F3.
After performing the three tests described above, the remaining genes in *C*_*two* − *way*_ are classified into *C*_*two* − *way* *combine*_, representing the two‐way combination effect group.

[B] Differential expression analysis for signature identification and interpretation

The second step was illustrated using a two‐way fixed‐effect model. After classifying the gene list into four categories, the iDAS framework performs differential gene expression analysis at each level of the factors for both main effects and interaction effects. In this case study, where factor 1 was cell state and factor 2 was phenotype, the process was as follows:

- Main Effect F1 (Cell state): Differential expression (DE) analysis was performed using limma^[^ ^12^ ^]^ on the main effect‐related gene signatures to identify cell markers characterizing each cell state.
- Main Effect F2 (Phenotype): DE analysis was performed using limma on the main effect‐related gene signatures to find signatures that are highly differentially expressed between the responding and non‐responding cell groups.
- Interaction Effect: For interaction effect, cell state–specific responding or non‐responding signatures were of primary interest. First, the top 10 marker genes of each cell state in the interaction signatures were identified. Then, Differential expression (DE) analysis was then repeated using these marker genes across the two phenotypes to determine whether any cell state marker genes were also differentially expressed between the phenotype groups. Genes specifically expressed in one phenotype within a given cell state were subsequently selected and designated as cell state–specific responding or non‐responding markers.

In sum, the iDAS R package allows users to apply either a linear regression model using the lm function in stats package or a linear mixed‐effects model using the lmer function from the lme4 package. For all hypothesis tests, users can choose to use either raw p‐values or adjusted p‐values cut off to identify whether a gene is associated with a factor of interest. In the first step, where genes are classified as associated or not associated with any factor, iDAS also provides an option to use a p‐value quantile cutoff. This is a user‐defined percentage value that sets the significance threshold based on the distribution of full model p‐values or adjusted p‐values (Figure S11, Supporting Information).

### Differential Expression Analysis using Limma

Pseudobulk expression matrices were generated by aggregating single‐cell data across cell states and treatment phenotypes. A linear model was constructed for each gene using the design formula ∼ cell state × treatment phenotype, incorporating both main effects and their interaction term. Top differentially expressed genes were identified for each model coefficient (cell state, treatment phenotype, and their interaction) using topTable, with an adjusted p‐value threshold of < 0.01. Genes were assigned to each factor based on their significance with respect to the corresponding model coefficient.

### Differential Abundance Analysis using Milo

Milo was used to perform differential abundance (DA) analysis on the single‐cell dataset to identify the most significant DA groups and exclude insignificant ones for further iDAS analysis. Following the official Milo guidelines, this analysis involved several steps: creating a Milo object, constructing a KNN graph with 30 PCA‐reduced dimensions, defining representative neighborhoods using the same k value (k = 10) used in building the KNN graph, counting cells in neighborhoods, building a design matrix, and conducting differential abundance testing, with all other parameters set to default. We then extracted the DA neighborhoods with a false discovery rate (FDR) of ≤0.2 and a log_2_ fold change (log_2_FC) of ≥5. This approach ensured that it was focused on the most relevant DA groups for our subsequent analysis.

### Differential Abundance Analysis using DA‐Seq and Scissor

DA‐seq with default parameters was applied to assign a differential abundance score to each cell, were selected asand selected cells with scores above 0.96 or below ‐0.96 differentially abundant cells. For Scissor, pseudobulk data were first created for each sample, using the patient‐level phenotype as the sample phenotype. Scissor analysis was then performed using the binomial approach with alpha = 0. As a result, all cells were classified as either responding or non‐responding cells.

### Apply iDAS to Pozniak2024 Single Cell Data

In this case study, the DA cells identified by Milo were used for the iDAS analysis. To apply iDAS with two‐way fix effect modelcell state and the patient’s immunotherapy response phenotype were taken as the two main effects. To avoid the high false discovery rate commonly associated with single‐cell analysis, usually due to a large number of replicates and violation of independence assumptions that can lead to false signals, pseudobulk samples were created instead of directly using single cells in the analysis. For each sample, the average gene expression per cell state was calculated. iDAS analysis was performed on the log2 transformed pseudobulk matrix. he iDAS model was used to identify the main effect signatures that are differentially expressed in cell states (F1), treatment phenotypes (F2), and the interaction effect signatures related to both factors (F1xF2). To apply iDAS with a three‐way random effect model, cell states, patient immunotherapy response phenotypes, and sample pre/on treatment status were used as the three main effect factors, with patient ID as the random effect factor.

### Hallmark Pathway Enrichment Analysis

To perform enrichment analysis on gene expression data, the bitr function from the clusterProfiler package was employed, which translates gene symbols to Entrez IDs using the org.Hs.eg.db database. Human gene set data were retrieved from msigdbr, specifically focusing on the Hallmark gene set collection.

For each set of positive markers (log_2_FC > 0) associated with a cell state or treatment phenotype, it was first assessed whether the subset contained more than five genes to proceed with enrichment analysis. If this criterion was met, the enricher function was applied to identify significantly enriched gene sets based on the translated Entrez IDs, using a minimum gene set size of five to ensure validity. The enriched gene sets were then visualized using the dotplot function from the same package, with plots titled according to their corresponding category.

### Bulk Deconvolution with BayesPrism

To demonstrate that the gene signatures identified by iDAS are transferable to external bulk datasets, cell states within the bulk dataset were first identified. The official guidelines for applyingto apply BayesPrism ^[^ ^34^ ^]^ for deconvolution of the bulk data into the corresponding cell states identified in the single‐cell data were followed. Genes with expression in fewer than five cells in the scRNA‐seq matrix were removed, and only protein‐coding genes were selected for the deconvolution analysis. All other parameters were set to their default values.

### Evaluation

General linear regression models were built to predict the response status or cell states of single‐cell pseudobulk samples or external bulk samples using the signatures. A nested 10 cross‐validation was performed using the nestcv.glmnet function from the nestedcv package and the generalized linear model was constructed with the glmnet package.

### Spatial Analysis with In‐Silico Spatially Resolved Transcriptomics Data on TCGA Breast Cancer

The GHIST prediction results, applied to a set of TCGA breast cancer dataset, were used. A set of samples focused on the HER2+ breast cancer subtype from TCGA‐BRCA was selected,  HER2+ patients were identified based on a positive entry in the “lab_proc_her2_neu_immunohistochemistry_receptor_status” metadata column, resulting in 92 TCGA samples with corresponding matched H&E images. The in‐silico prediction was described in detailed from GHIST paper.^[^ ^35^ ^]^ After filtering out NA values in the AJCC pathological stage, only ER/PR positive (ER+/PR+; n = 54) and ER/PR negative (ER−/PR−; n = 22) in‐silico spatially resolved transcriptomics samples were selected. In total, 274 genes were predicted using the GHIST package. scFeatures was then applied to calculate the nearest neighbor correlation (nnCorrelation) between these genes and their most adjacent neighboring cells, resulting in a nearest neighbor correlation matrix of 274 genes across 76 samples. the nearest neighbor correlation was then explored with the factor of ER/PR status and the AJCC pathological stage.

## Conflict of Interest

The authors declare no conflict of interest.

## Author Contributions

J.Y. conceived, designed and funded the study with input from P.Y. and Y.L., L.Y. completed the analysis and with guidance from Y.L., J.Y. and P.Y. The modelling and R package was done by L.Y. with support from X.X. All authors wrote, reviewed and approved the manuscript.

## Supporting information



Supporting Information

Supporting Information

## Data Availability

The data that support the findings of this study are available in the supplementary material of this article.
