# Supplementary material for: Interpretable Differential Abundance Signature (iDAS)
Source: Small Methods. 2025 May 27;10(2):e2500572. doi: 10.1002/smtd.202500572 (PMC12825360; doi:10.1002/smtd.202500572)
Supplement: Supplementary file 1 — Supporting Information [file SMTD-10-e2500572-s001.docx]

**Supporting Information**

**Title: Interpretable Differential Abundance Signature (iDAS)**

*Lijia Yu, Yingxin Lin, Xiangnan Xu, Pengyi Yang, Jean YH Yang**

**Supplementary File1**

Results from the melanoma dataset, including iDAS identified genes associated with main effects, interaction effects, and additive effects for both two-factor and three-factor models. Also includes cell state hallmark enrichment results for the two-factor and three-factor models.

**Supplementary File2**

Results from the two-factor differential expression analysis using limma. This includes all gene-level testing results for cell state effects, treatment phenotype effects, and their interaction, based on the pseudobulk melanoma dataset.

**Supplementary Figures**


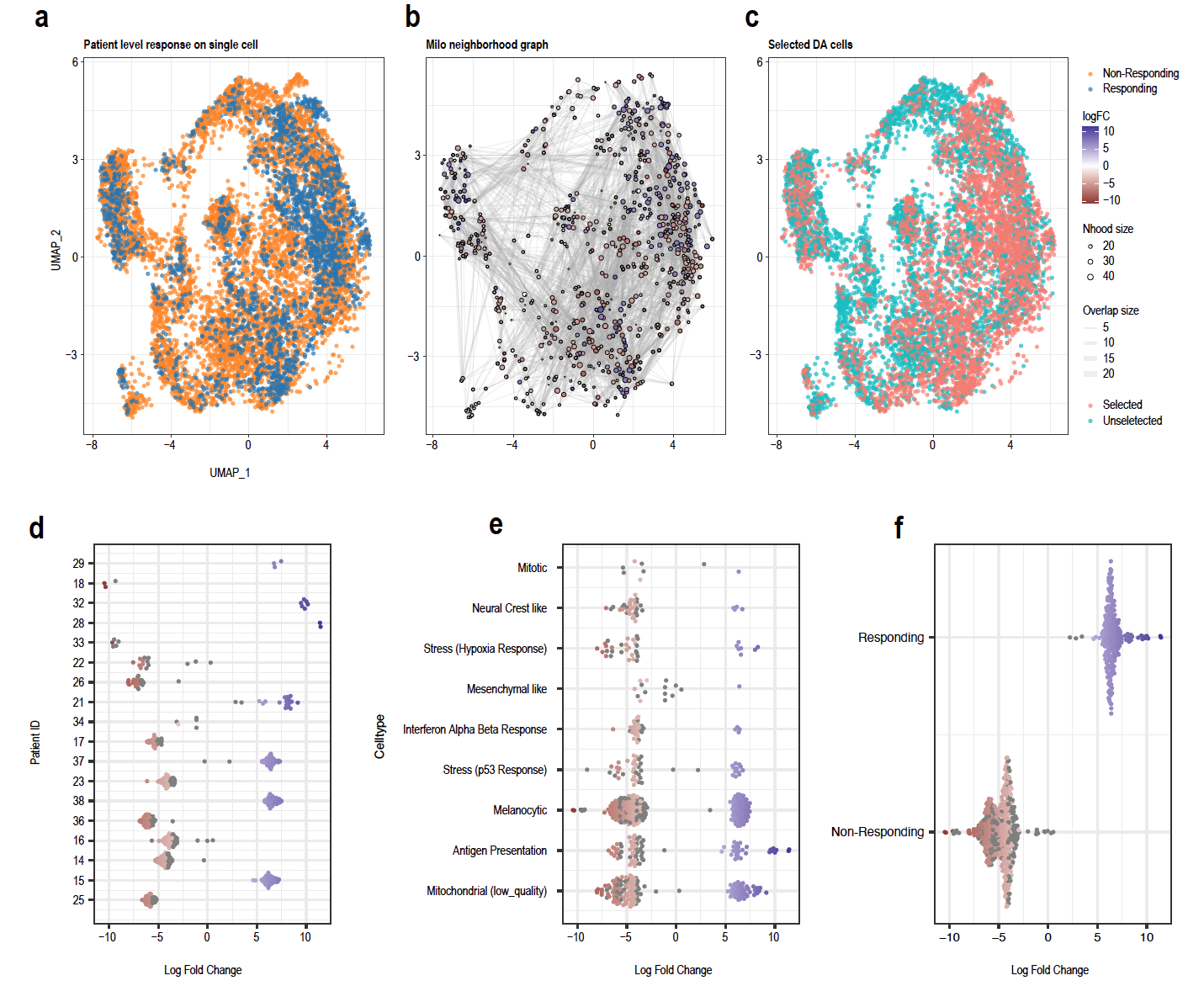


**Supplementary Figure S1:** Using miloR to identify differentially abundant cells. (a) UMAP plot of patient-level response in the single-cell dataset. (b) miloR identified differentially abundant (DA) neighborhood with a Spatial FDR ⩽ 0.2. (c) UMAP plot of selected single cells from miloR DA neighborhood groups with a Spatial FDR ⩽ 0.2 and log_2_ fold change ⩾ 5. (d) Beeswarm plot visualizes the distribution of log_2_ fold changes across neighborhood annotations of patient ID. (e) Beeswarm plot shows the distribution of log_2_ fold changes across cell states. (f) Beeswarm plot illustrates the distribution of log_2_ fold changes across phenotype.


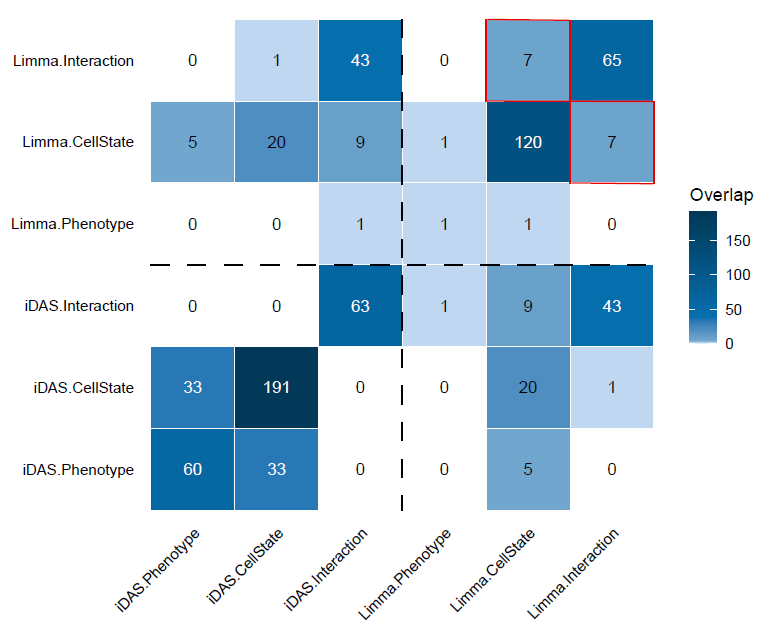


**Supplementary Figure S2:** Comparison of gene classification between iDAS and classic differential expression analysis. Heatmap showing the overlap of significant genes grouped by iDAS and limma into phenotype-, cell state-, and interaction-associated categories. Additive effect genes identified by iDAS were reassigned to the corresponding main effect categories (phenotype or cell state) for comparability. Red boxes highlight genes that are simultaneously considered with both interaction- and main-effect-associated, illustrating the ambiguity in effect attribution without structured classification.


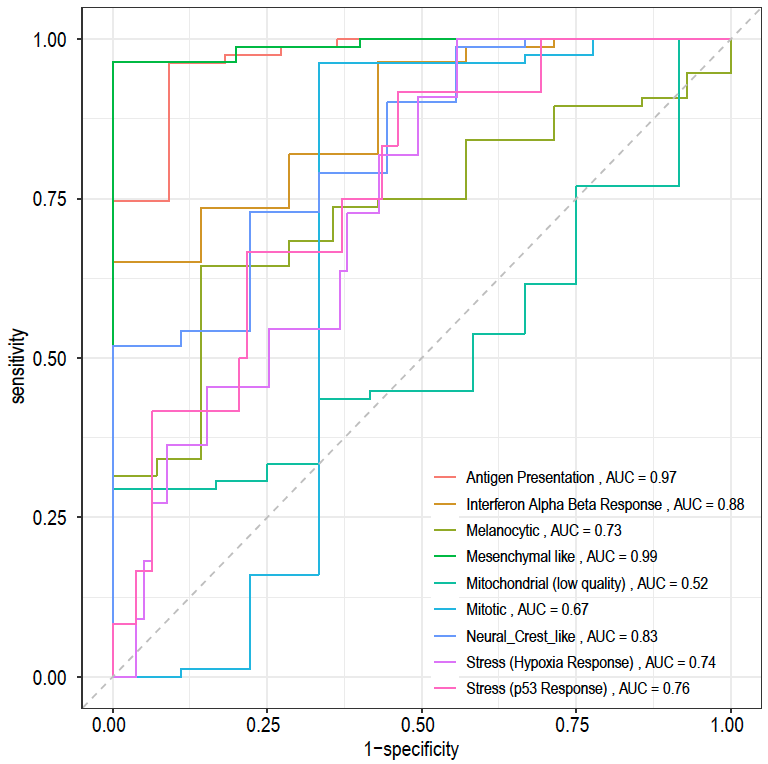


**Supplementary Figure S3:** ROC curves for cell state-specific gene signatures. The AUC values for each signature are as follows: Antigen Presentation (AUC = 0.97), Interferon Alpha Beta Response (AUC = 0.88), Melanocytic (AUC = 0.73), Mesenchymal-like (AUC = 0.99), Mitochondrial (low quality, AUC = 0.52), Mitotic (AUC = 0.67), Neural Crest-like (AUC = 0.83), Stress (Hypoxia Response, AUC = 0.74), and Stress (p53 Response, AUC = 0.76). The dashed line represents the line of no discrimination (AUC = 0.5).


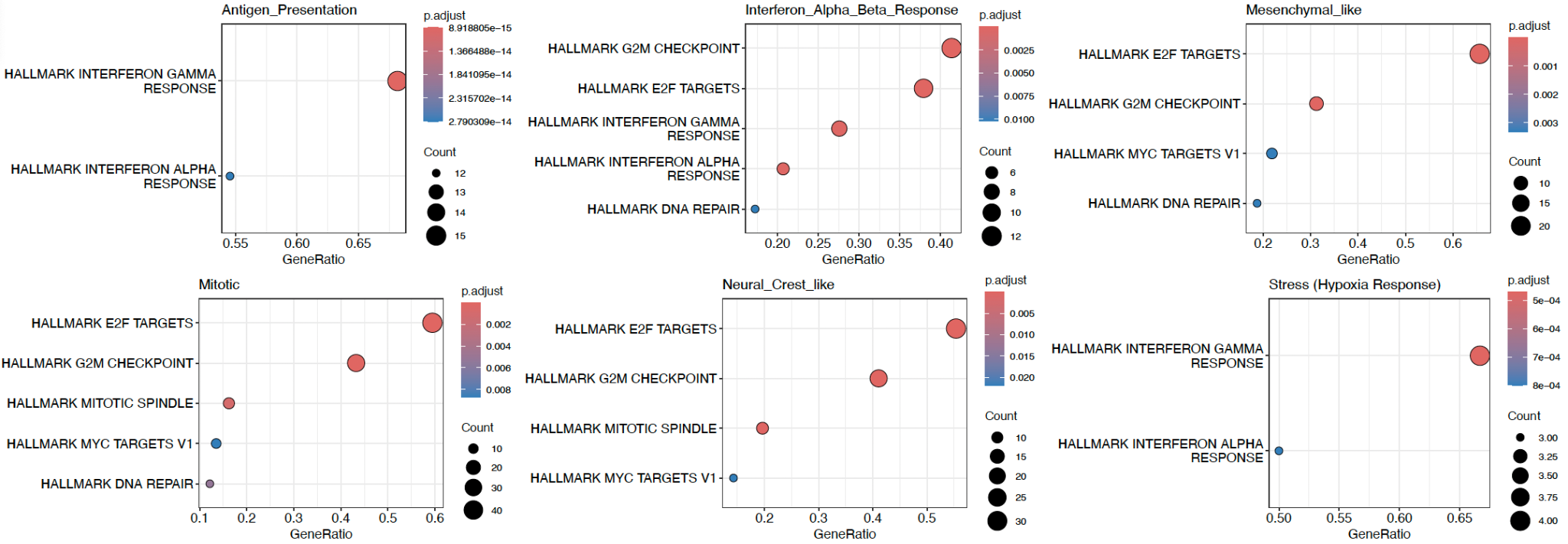


**Supplementary Figure S4:** Hallmark gene set enrichment analysis of cell state-specific signatures (positive marker) obtained from the two-way iDAS model. Hallmark gene enrichment on six cell states. The gene ratio for each hallmark is plotted along the x-axis, and the size of each dot represents the count of genes in the corresponding gene set. Colors indicate the adjusted p-values, with a gradient from red (most significant) to blue (least significant).


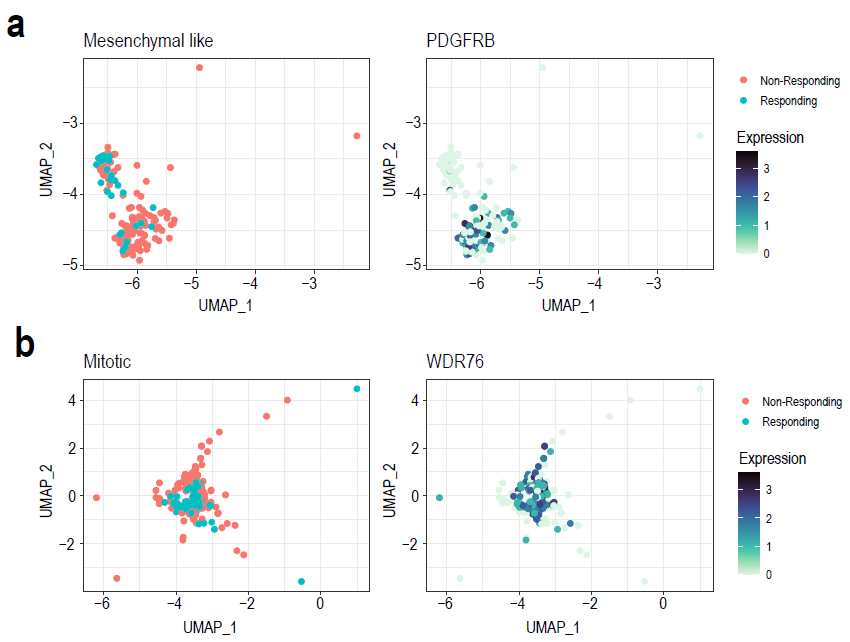


**Supplementary Figure S5:** iDAS identifies the interaction effect signatures between phenotype and cell states. Cell state specific phenotype signatures were selected from mesenchymal-like cells and mitotic cells. (a,b) UMAP plots of mesenchymal-like cells and mitotic cells, respectively, with patient-level phenotype colored in each single cell in left panels and gene expression values of *PDGFRB* and *WDR76* within their respective cell states in right panels.


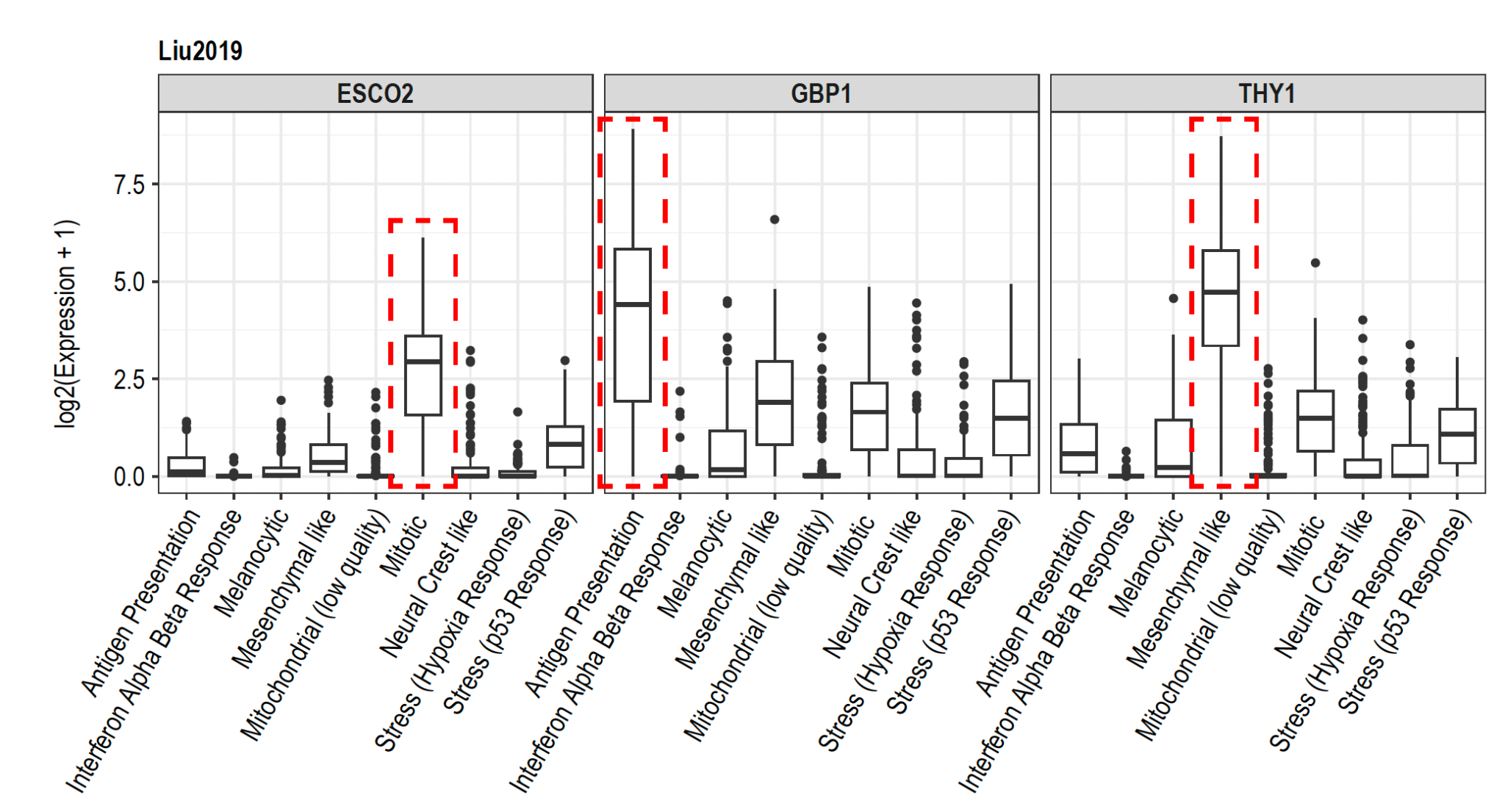


**Supplementary Figure S6:** The cell state main effect signatures validation on external bulk data (Liu2019). The box plot shows the expression of genes *ESCO2*, *GBP1* and *THY1*, mapped onto cell state specific deconvolutions of external bulk RNA dataset. Consistent with the observations in Figure 2c, these genes exhibit high expression in particular cell states, underscoring their role as specific markers across different datasets.


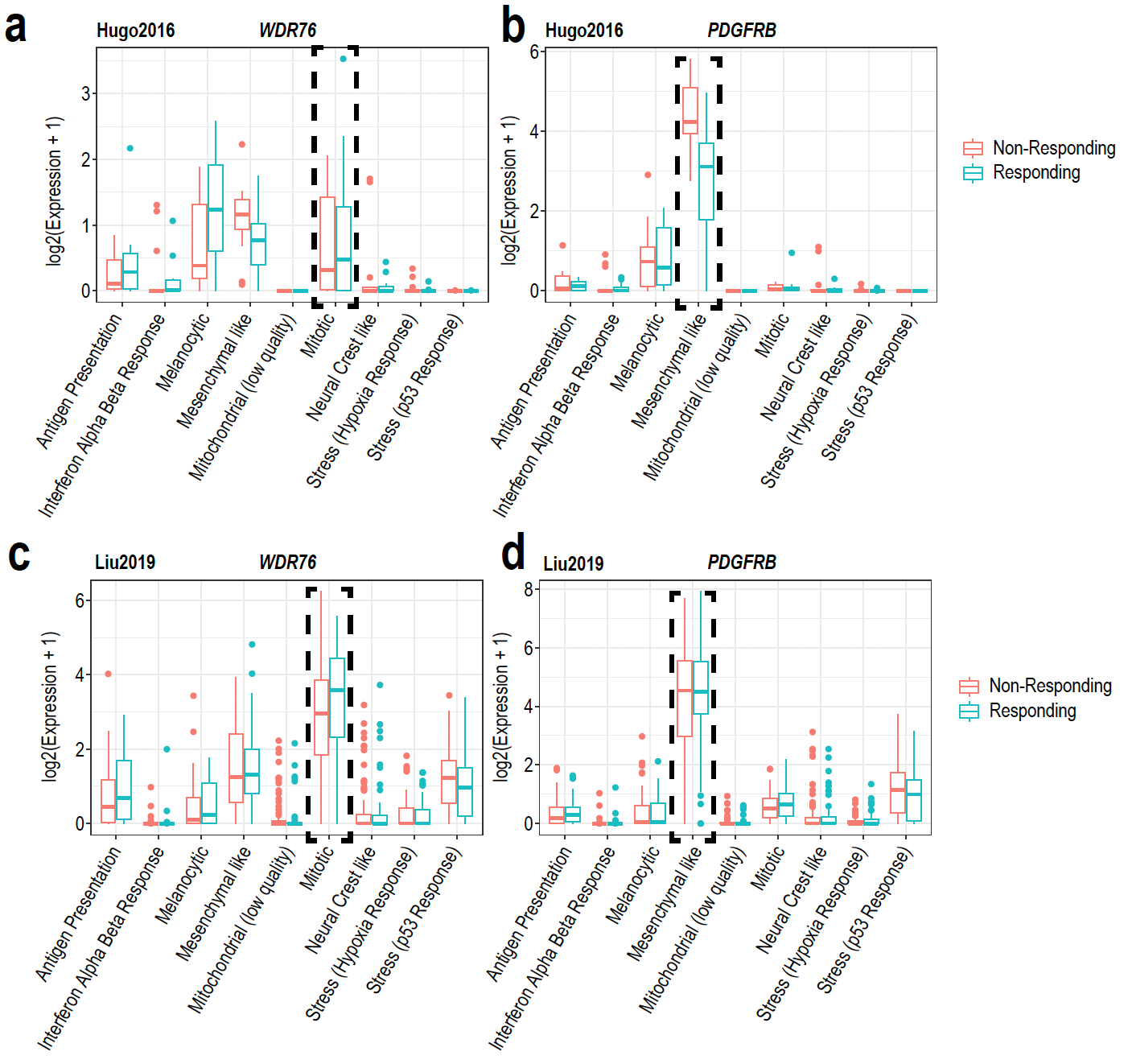


**Supplementary Figure S7:** The interaction effect signatures, such as cell state specific responding signatures, are transferable to external bulk data. (a,c) Box plot showing *WDR76* gene expression levels in deconvoluted cell states from Hugo2016 and Liu2019 bulk samples. (b,d) Box plot showing *PDGFRB* gene expression levels in deconvoluted cell states from Hugo2016 and Liu2019 bulk samples. The colors indicate the phenotype of the bulk samples.


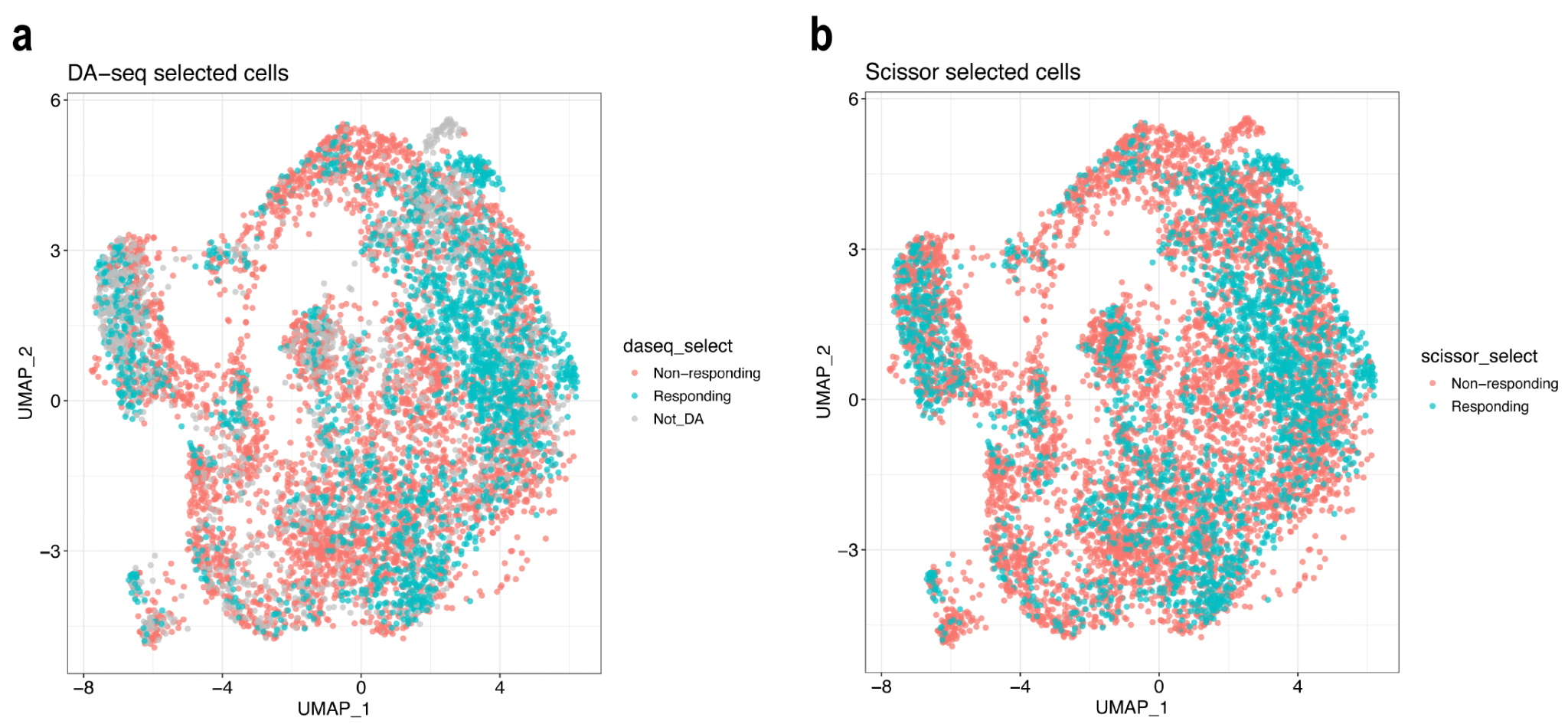


**Supplementary Figure S8:** UMAP plots of selected single cells from DA-seq (a) and Scissor (b). DA-seq includes responding cells, non-responding cells, and filtered non-DA cells, while Scissor selects all cells as either responding or non-responding.


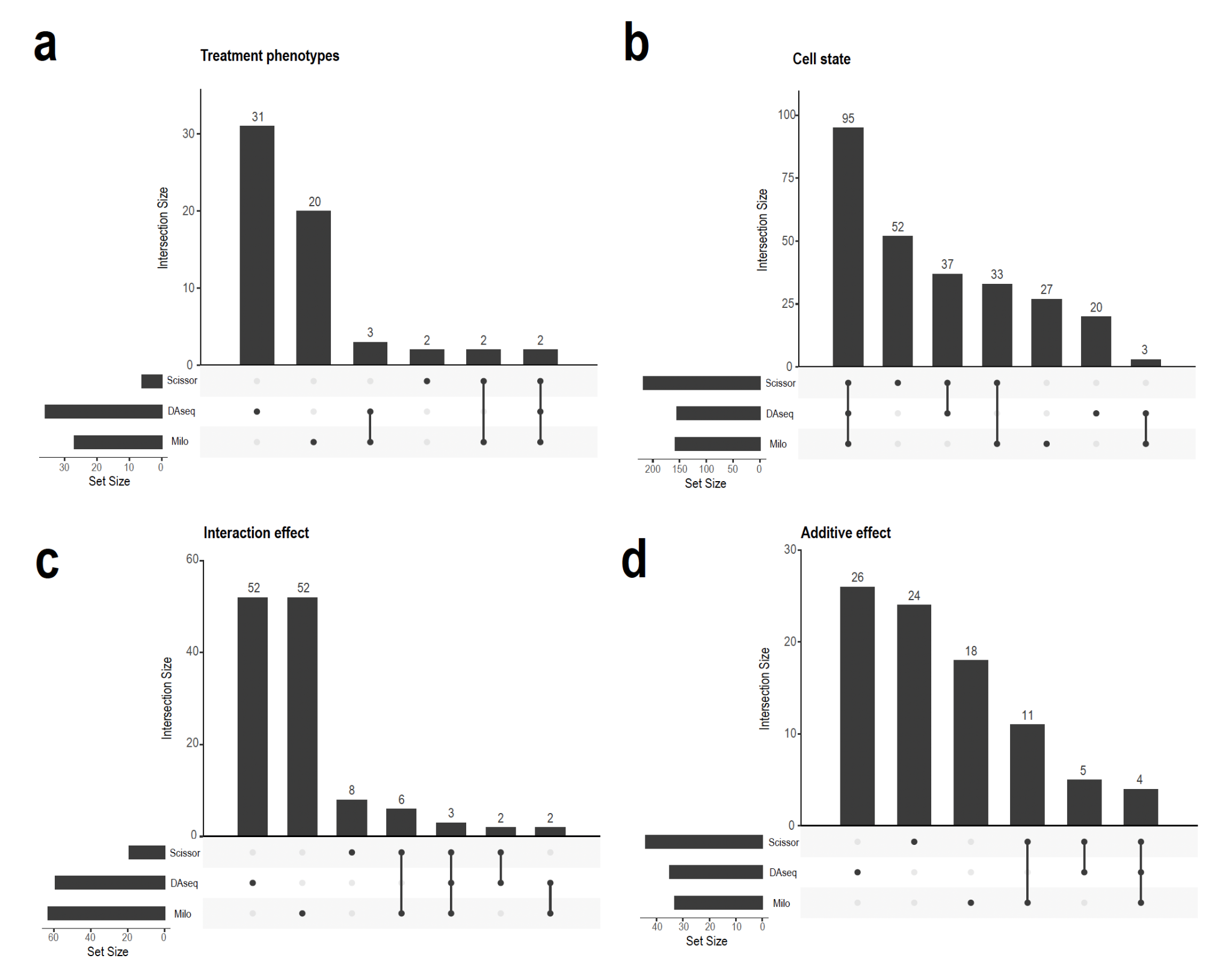


**Supplementary Figure S9:** UpSet plots showing gene set overlaps across three different DA + iDAS pipelines. (a) Intersection of treatment phenotype-associated gene sets. (b) Intersection of cell state-associated gene sets. (c) Intersection of interaction effect-associated gene sets. (d) Intersection of additive effect-associated gene sets. Each panel compares gene lists generated by different differential abundance (DA) detection methods combined with iDAS.


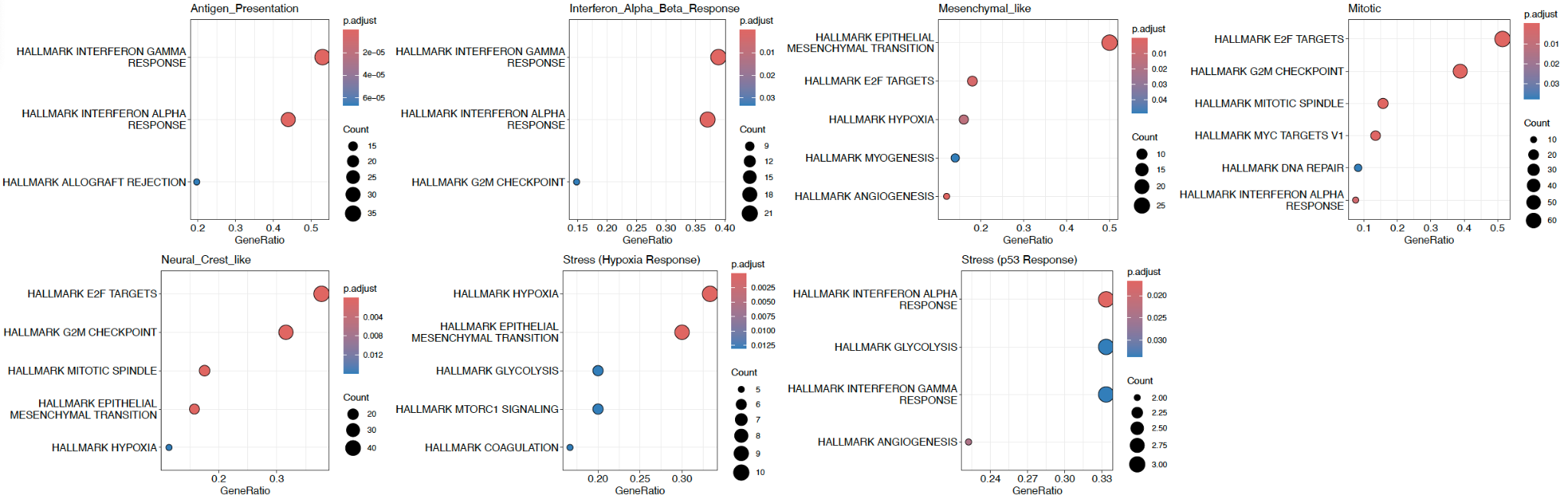


**Supplementary Figure S10:** Hallmark gene set enrichment analysis of cell state-specific signatures (positive markers) obtained from the three-way iDAS model. Hallmark gene enrichment on seven cell states. The gene ratio for each hallmark is plotted along the x-axis, and the size of each dot represents the count of genes in the corresponding gene set. Colors indicate the adjusted p-values, with a gradient from red (most significant) to blue (least significant).


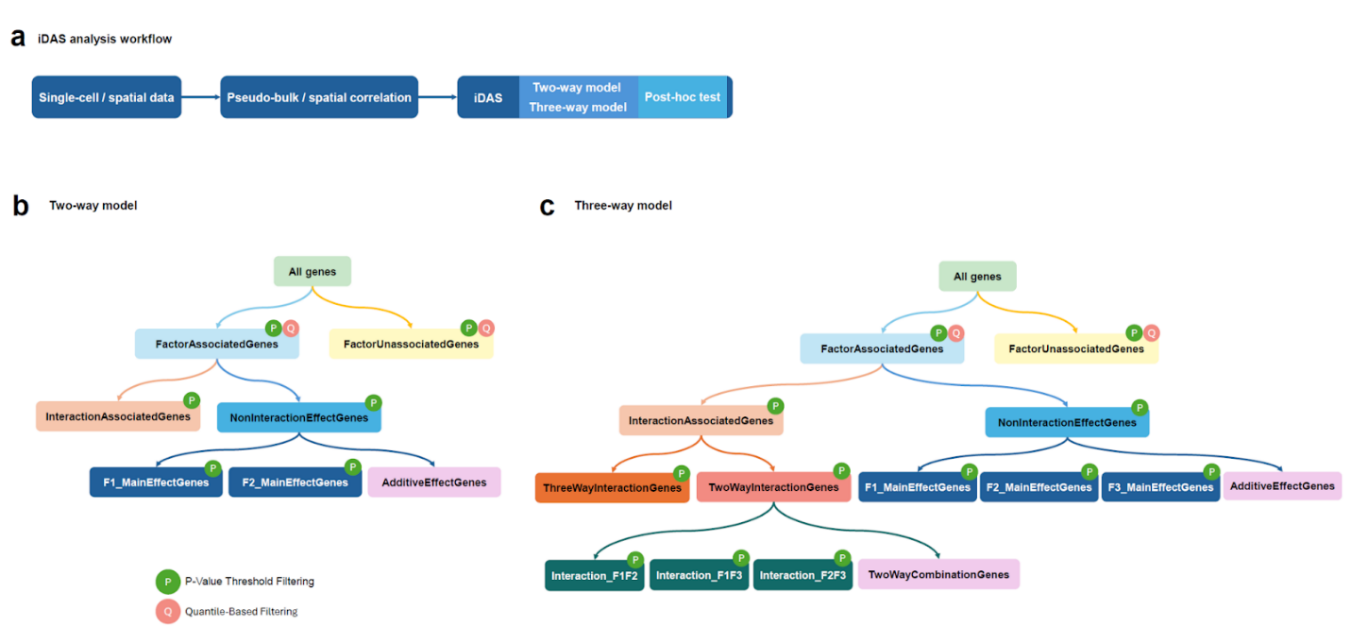


**Supplementary Figure S11:** iDAS analysis workflow and the “nested” ANOVA test to classify genes into different categories. (a) iDAS analysis workflow. (b) two-way model. (c) three-way model.
